# A CT Lesion Foundation Model for Non‐Invasive Assessment of Hepatocellular Carcinoma Aggressiveness and Prediction of Recurrence Risk

**DOI:** 10.1002/advs.78587

**Published:** 2026-11-01

**Authors:** Wei Xia, Zhehan Shen, Mengsi Li, Jianxin Lu, Ji Xia, Yang Cheng, Wenzheng Li, Shunjie Dong, Lin Deng, Ruokun Li

**Affiliations:** ^1^ Department of Radiology & Faculty of Medical Imaging Technology Ruijin Hospital College of Health Science and Technology Shanghai Jiao Tong University School of Medicine Shanghai China; ^2^ Department of Radiology Xiangya Hospital Central South University Changsha China

**Keywords:** artificial intelligence, computer tomographic, deep learning, hepatocellular carcinoma, risk assessment

## Abstract

Hepatocellular carcinoma (HCC) remains a leading cause of cancer‐related mortality worldwide, with high recurrence rates following curative resection. Accurate non‐invasive assessment of tumor aggressiveness and recurrence risk is critical for surgical decision‐making and postoperative surveillance, yet remains challenging with conventional imaging modalities. Here, we developed a CT lesion foundation model (LFM), trained via self‐supervised learning on 32,120 generic lesion and 6,387 HCC‐specific CT patches to capture transferable intra‐lesional imaging phenotypes. Across one internal and two external validation cohorts, the sequentially pretrained LFM (LFM‐Seq) achieved area under the curve values of 0.916, 0.846, and 0.920 for microvascular invasion (MVI) assessment, and 0.833, 0.885 and 0.795 for pathological grade assessment. The model remained robust under scenarios of limited training data and tumor localization perturbations. Furthermore, LFM‐Seq‐derived risk scores stratified recurrence‐free survival (RFS), yielding an externally validated C‐index of 0.701, and remained independently associated with RFS after adjusting for clinicopathological factors. Exploratory biological interpretability analyses showed that LFM‐Seq‐derived risk phenotypes were associated with suppressed lymphocyte‐related immune programs. These results suggest that CT lesion foundation models can support non‐invasive and robust characterization of HCC aggressiveness, recurrence risk stratification and discovery of interpretable prognostic imaging biomarkers.

## Graphical abstract

A computed tomography lesion foundation model, built by sequential self‐supervised pretraining on generic and hepatocellular carcinoma‐specific lesion images, non‐invasively assesses microvascular invasion and pathological grade from contrast‐enhanced CT and stratifies postoperative recurrence risk. Multicenter validation demonstrates robust performance, and imaging‐genomic analysis links high‐risk phenotypes to suppressed lymphocyte‐mediated immune programs.

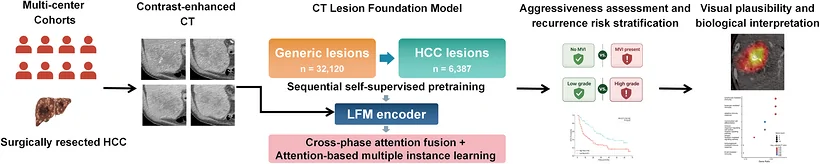

## 1 Introduction

Hepatocellular carcinoma (HCC) is the most common primary liver malignancy and remains a leading cause of cancer‐related mortality worldwide [1]. Despite advances in surveillance and surgical technique, long‐term outcomes after curative‐intent therapy remain constrained by high postoperative recurrence rates [2, 3, 4]. Microvascular invasion (MVI) and high pathological grade (Edmondson‐Steiner grade 3/4) represent two key pathological hallmarks of aggressive HCC biology. MVI reflects occult vascular dissemination and inherent invasive potential, whereas high pathological grade indicates poor differentiation and malignant cellular phenotype. Collectively, these two features are increasingly recognized as high‐risk histopathological markers associated with early recurrence and poor survival [5, 6, 7].

Current clinical assessment of MVI and pathological grade relies on postoperative histopathological examination, which limits its utility for preoperative risk stratification and individualized surgery planning [6, 7]. Needle biopsy can provide histological information prior to treatment; however, its clinical value is constrained by sampling error, potential complications and substantial intratumoral spatial heterogeneity within HCC [2, 8]. Contrast‐enhanced computed tomography (CECT) is routinely used for HCC detection, staging, treatment planning and response evaluation, and standardized reporting systems have improved consistency in imaging interpretation [2, 9, 10]. Nevertheless, visual assessment of aggressive or prognostic imaging features remains subjective and is subject to inter‐reader variability, particularly when features are subtle, borderline or heterogeneously distributed within the tumor [10, 11], highlighting the need for objective and quantitative image analysis methods.

With the rapid advancement of artificial intelligence, radiomics and deep learning have been explored as quantitative approaches for preoperative HCC characterization and prognostication [12, 13, 14, 15, 16]. Radiomics can extract high‐dimensional handcrafted features from medical images, however, it relies heavily on preprocessing choices, segmentation reproducibility, feature engineering and model selection [17]. Task‐specific deep learning models can learn image features directly from data, but they often require large annotated cohorts and may generalize poorly across institutions, scanners and imaging protocols [14, 18, 19]. In addition, many prior studies have focused primarily on diagnostic discrimination, whereas robustness under clinically relevant perturbations, data‐efficient learning and biological interpretability remain insufficiently explored [12, 14, 18].

The emergence of vision foundation models offers a new paradigm for medical image analysis leveraging large‐scale self‐supervised pretraining to learn transferable features that can be adapted to downstream tasks with fewer labels [20, 21, 22]. Self‐supervised vision transformer frameworks have shown strong capacity for learning transferable features and fine‐grained local features, and have recently been adapted in pathology and radiology foundation model studies [23, 24, 25, 26, 27]. However, many existing imaging foundation models have been developed from organ‐level, whole‐image or broad anatomical contexts [20, 21, 22, 27]. By contrast, CT imaging differences corresponding to varying HCC aggressiveness are primarily reflected in subtle lesion‐level features, including heterogeneous intratumoral density and enhancement, phase‐specific enhancement patterns, tumor margin morphology, and intratumoral hypodense necrotic areas. Lesion‐centric feature learning may therefore be particularly valuable for tumors whose clinically relevant biology is encoded in localized imaging patterns [10, 21, 28]. A recent lesion‐centric CT foundation model has demonstrated the feasibility of learning generalizable lesion features from annotated CT patches [21]. However, whether such general lesion‐level features can be optimally adapted to multiphase HCC imaging and to biologically relevant endpoints including MVI, pathological grade and postoperative recurrence remains insufficiently explored. In particular, HCC aggressiveness may be encoded in subtle intra‐lesional enhancement heterogeneity and local contextual patterns across arterial and portal venous phases, which may not be fully captured by features learned from generic lesions alone. These considerations motivated a sequential pretraining strategy, first learning general lesion representations from a large and diverse collection of lesion images, followed by lightweight adaptation to HCC‐specific multiphase images, to preserve broad lesion knowledge while aligning features with HCC‐specific patterns, analogous to a curriculum from general to domain‐specific representations.

In this study, we developed a lesion foundation model (LFM) pretrained on 32 120 generic lesion patches and 6387 HCC‐specific CT patches using a self‐supervised DINOv2 framework, aiming to learn transferable intra‐lesional imaging features from CECT (Figure 1A,B). To enable patient‐level characterization from variable‐length lesion patch sets, we integrated LFM with attention‐based multiple instance learning (ABMIL), allowing simultaneous assessment of MVI and pathological grade while learning whole‐tumor features of HCC aggressiveness (Figure 1C). Leveraging the learned whole‐tumor features, we constructed elastic‐net Cox models to generate LFM‐derived recurrence‐free survival (RFS) risk scores and stratify postoperative recurrence risk (Figure 1D). Finally, occlusion‐based visual interpretation and genomic association analyses were performed to examine the spatial plausibility of aggressiveness evaluation and biological relevance of LFM‐derived risk phenotypes (Figure 1E). Collectively, our results establish LFM as a framework for non‐invasive characterization of HCC aggressiveness and for discovery of biologically interpretable imaging biomarkers of recurrence risk.

**FIGURE 1:**
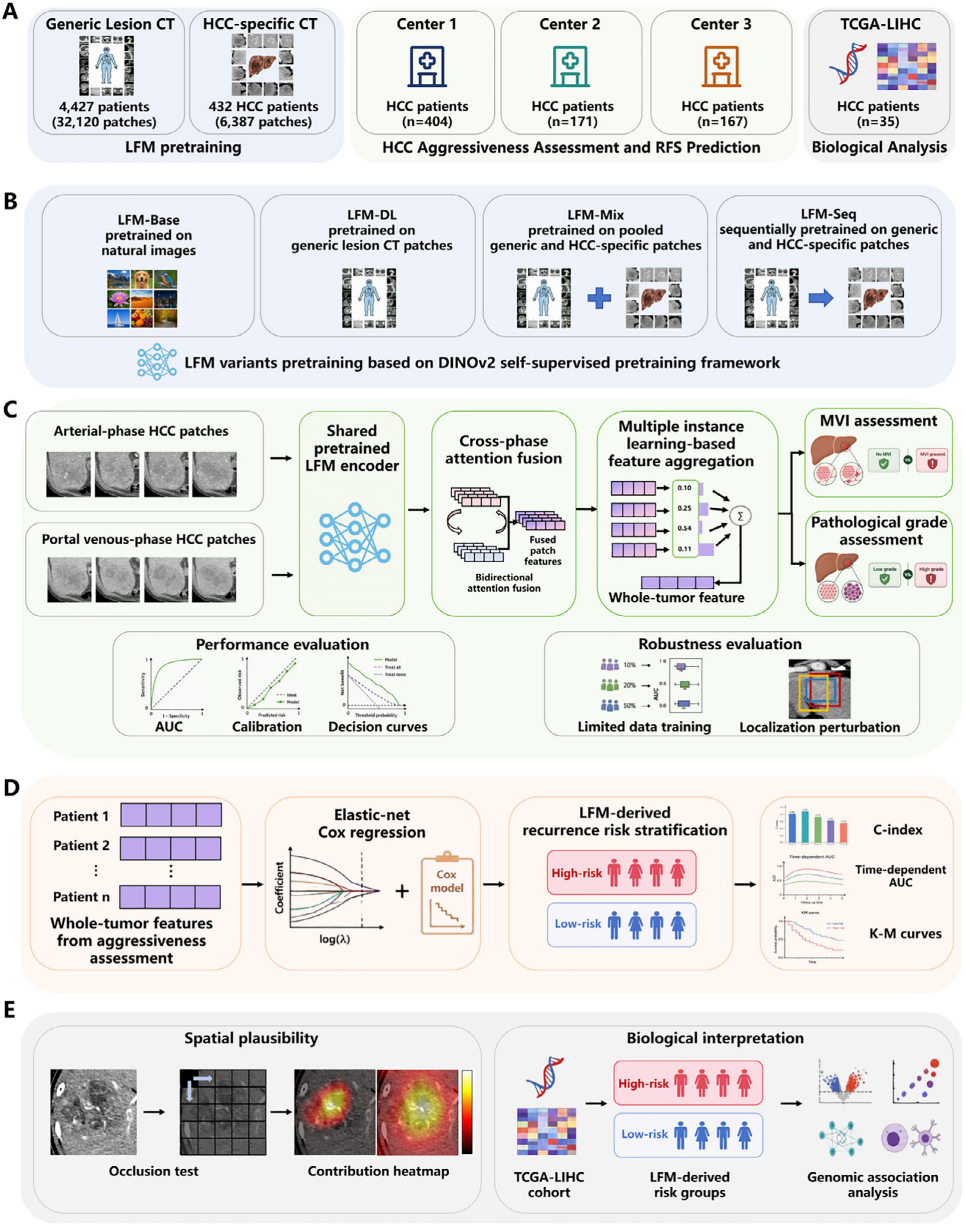
Study design and overview of workflow. (A) Study design and dataset‐task assignment. Generic lesion CT patches from DeepLesion and HCC‐specific CT patches from PLC, WAW and MDA datasets were used for lesion foundation model (LFM) pretraining. Multicenter HCC cohorts from Centers 1–3 were used for HCC aggressiveness assessment and recurrence‐free survival (RFS) prediction, and the TCGA‐LIHC cohort with matched CT imaging and transcriptomic data was used for biological interpretation. (B) Construction of LFM variants. LFM‐Base was the official DINOv2 model pretrained on natural images, LFM‐DL was pretrained on generic lesion CT patches, LFM‐Mix was pretrained on pooled generic lesion and HCC‐specific CT patches, and LFM‐Seq was sequentially pretrained from generic lesion CT patches to HCC‐specific CT patches. (C) LFM‐based HCC aggressiveness assessment. Arterial‐phase and portal venous‐phase HCC patches were processed by a shared pretrained LFM encoder, followed by cross‐phase attention fusion and multiple instance learning to aggregate whole‐tumor features for microvascular invasion (MVI) assessment and pathological grade assessment. Model performance was evaluated using discrimination, calibration and decision‐curve analyses, and robustness was assessed under limited training data and tumor‐localization perturbation settings. (D) RFS prediction using LFM‐derived whole‐tumor features. Whole‐tumor features learned from HCC aggressiveness assessment were used to train an elastic‐net Cox regression model, generate recurrence risk scores and stratify patients into high‐risk and low‐risk groups. Model performance was evaluated by C‐index, time‐dependent AUC and Kaplan‐Meier analysis. (E) Visual and biological interpretation analysis. Spatial plausibility was assessed using occlusion‐based heatmaps, and biological interpretation was performed by linking LFM‐derived risk groups with TCGA‐LIHC transcriptomic data.

## 2 Results

### 2.1 Characteristics of the Included Cohorts

The data inclusion and exclusion flow diagram of this study is illustrated in Figure S1. For LFM development, pretraining datasets were constructed from 32 120 generic lesion CT patches derived from the DeepLesion dataset and 6,387 HCC‐specific CT patches collected from public HCC datasets.

A total of 742 patients with pathologically confirmed HCC were enrolled from three independent centers for supervised downstream analyses. For aggressiveness assessment, Center 1 included 404 patients (median age 60.0 years [IQR: 52.0–67.0]; 80.9% male) and was divided into a training cohort (*n* = 263), used for model training and fine‐tuning, and an internal validation cohort (IVC; *n* = 141), used for internal evaluation. External validation cohort 1 (EVC1) from Center 2 included 171 patients (median age 59.0 years [IQR: 50.0–67.0]; 86.5% male), and external validation cohort 2 (EVC2) from Center 3 included 167 patients (median age 55.0 years [IQR: 48.0–65.5]; 83.8% male), which were used to externally validate model performance in aggressiveness assessment.

The prevalence of MVI was 32.2% (Center 1), 43.9% (Center 2), and 56.3% (Center 3). High pathological grade was present in 50.2%, 63.7%, and 52.1% of patients in Center 1, Center 2, and Center 3, respectively. The three cohorts showed significant differences in age, maximum tumor diameter, MVI status, pathological grade, and serum biomarkers including AFP, AST, GGT, and HBsAg, reflecting the heterogeneity of multi‐center clinical data (Table 1).

**TABLE 1:** Characteristics of patients with hepatocellular carcinoma in each center.

Characteristic	Center 1 (*n* = 404)	Center 2 (*n* = 171)	Center 3 (*n* = 167)	*p* value
Age (y)^a^	60.0 (52.0–67.0)	59.0 (50.0–67.0)	55.0 (48.0–65.5)	< 0.001
Sex				0.246
Male	327 (80.9%)	148 (86.5%)	140 (83.8%)	
Female	77 (19.1%)	23 (13.5%)	27 (16.2%)	
Maximum tumor diameter (cm)^a^	4.1 (2.7–6.4)	5.3 (3.7–9.0)	6.4 (4.1–9.7)	< 0.001
MVI				< 0.001
Present	130 (32.2%)	75 (43.9%)	94 (56.3%)	
Absent	274 (67.8%)	96 (56.1%)	73 (43.7%)	
Pathological grade				0.011
High‐grade	203 (50.2%)	109 (63.7%)	87 (52.1%)	
Low‐grade	201 (49.8%)	62 (36.3%)	80 (47.9%)	
AFP (ng mL^−1^)				< 0.001
> 200	106 (26.2%)	70 (40.9%)	74 (44.3%)	
≤ 200	298 (73.8%)	101 (59.1%)	93 (55.7%)	
ALT (IU L^−1^)				0.429
> 50	69 (17.1%)	37 (21.6%)	32 (19.2%)	
≤ 50	335 (82.9%)	134 (78.4%)	135 (80.8%)	
AST (IU L^−1^)				< 0.001
> 40	120 (29.7%)	77 (45.0%)	75 (44.9%)	
≤ 40	284 (70.3%)	94 (55.0%)	92 (55.1%)	
GGT (IU L^−1^)				0.035
> 60	147 (36.4%)	77 (45.0%)	53 (31.7%)	
≤ 60	257 (63.6%)	94 (55.0%)	114 (68.3%)	
HBsAg (IU mL^−1^)				0.007
≥ 0.05	291 (72.0%)	137 (80.1%)	139 (83.2%)	
< 0.05	113 (28.0%)	34 (19.9%)	28 (16.8%)	

*Note*: Unless otherwise indicated, data are numbers of patients, with percentages in parentheses.

Abbreviations: MVI, microvascular invasion; AFP, α‐fetoprotein; ALT, alanine aminotransferase; AST, aspartate aminotransferase; GGT, γ‐glutamyl transpeptidase; HBsAg, hepatitis B surface antigen; low pathological grade = Edmondson‐Steiner grade 1–2; High pathological grade = Edmondson‐Steiner grade 3–4. Differences between cohorts were compared using the Kruskal‐Wallis test for continuous variables and the Chi‐square test for categorical variables.

^a^ Data are medians, with interquartile ranges in parentheses.

The RFS prediction analyses were performed in EVC1 and EVC2, where complete postoperative recurrence follow‐up was available. EVC1 was used to develop the RFS prediction models, and EVC2 was used as an independent validation cohort. In EVC1, 131 events (76.6%) and 40 censored patients (23.4%) were observed; in EVC2, 106 events (63.5%) and 61 censored patients (36.5%) were observed. Median follow‐up was 56.0 months (95% CI, 41.0–60.0) in EVC1 and 41.0 months (95% CI, 32.0–47.0) in EVC2, respectively. The numbers of patients remaining at risk at 12, 24, and 36 months were 79, 40, and 20 in EVC1 and 91, 57, and 32 in EVC2.

### 2.2 Performance of MVI and Pathological Grade Assessment

We evaluated six models for non‐invasive assessment of HCC aggressiveness, including four LFM variants and two previous models. LFM‐Base served as the baseline foundation model without lesion‐level CT self‐supervised adaptation; LFM‐DL was pretrained on generic lesion CT patches from DeepLesion; LFM‐Mix was jointly pretrained on generic lesion and HCC‐specific CT patches; and LFM‐Seq was sequentially pretrained from generic lesions to HCC‐specific lesions. Radiomics and FMCIB [21] were included as handcrafted and 3D foundation model‐based baselines, respectively. Detailed diagnostic metrics for MVI and pathological grade assessment are summarized in Tables S1 and S2 and visualized in Figures 2 and 3.

**FIGURE 2:**
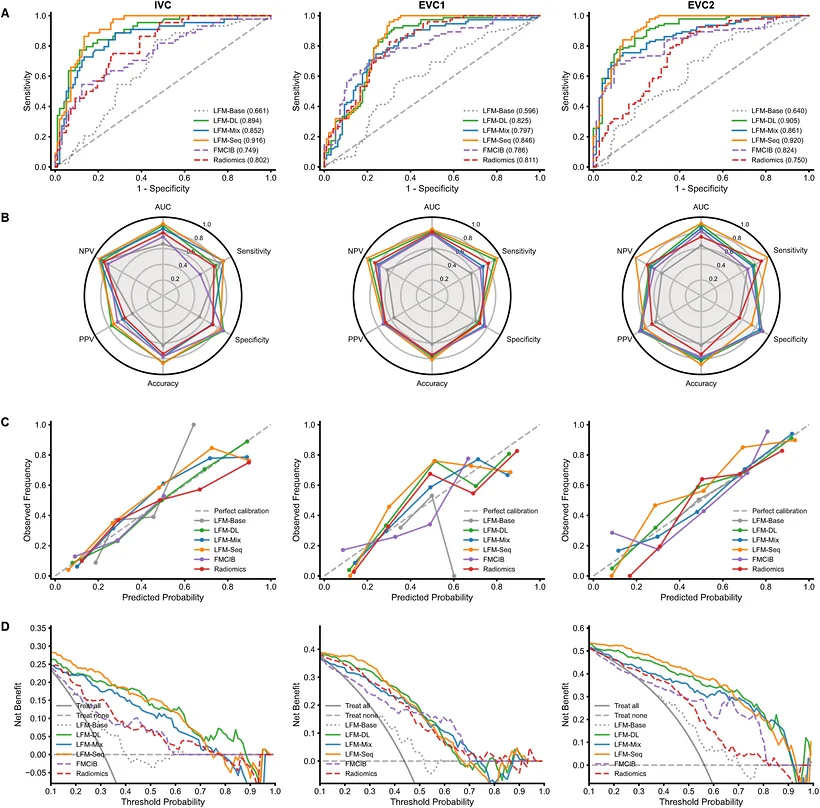
Performance of models for non‐invasive MVI assessment. (A) Receiver operating characteristic curves for MVI assessment in the internal validation cohort (IVC), external validation cohort 1 (EVC1), and external validation cohort 2 (EVC2). (B) Radar plots summarizing multidimensional assessment performance, including the area under the curve (AUC), sensitivity, specificity, accuracy, positive predictive value (PPV), and negative predictive value (NPV). (C) Calibration curves comparing model‐assessed MVI probabilities with observed frequencies across the three cohorts. (D) Decision curve analysis showing the net benefit of each model across a range of threshold probabilities.

**FIGURE 3:**
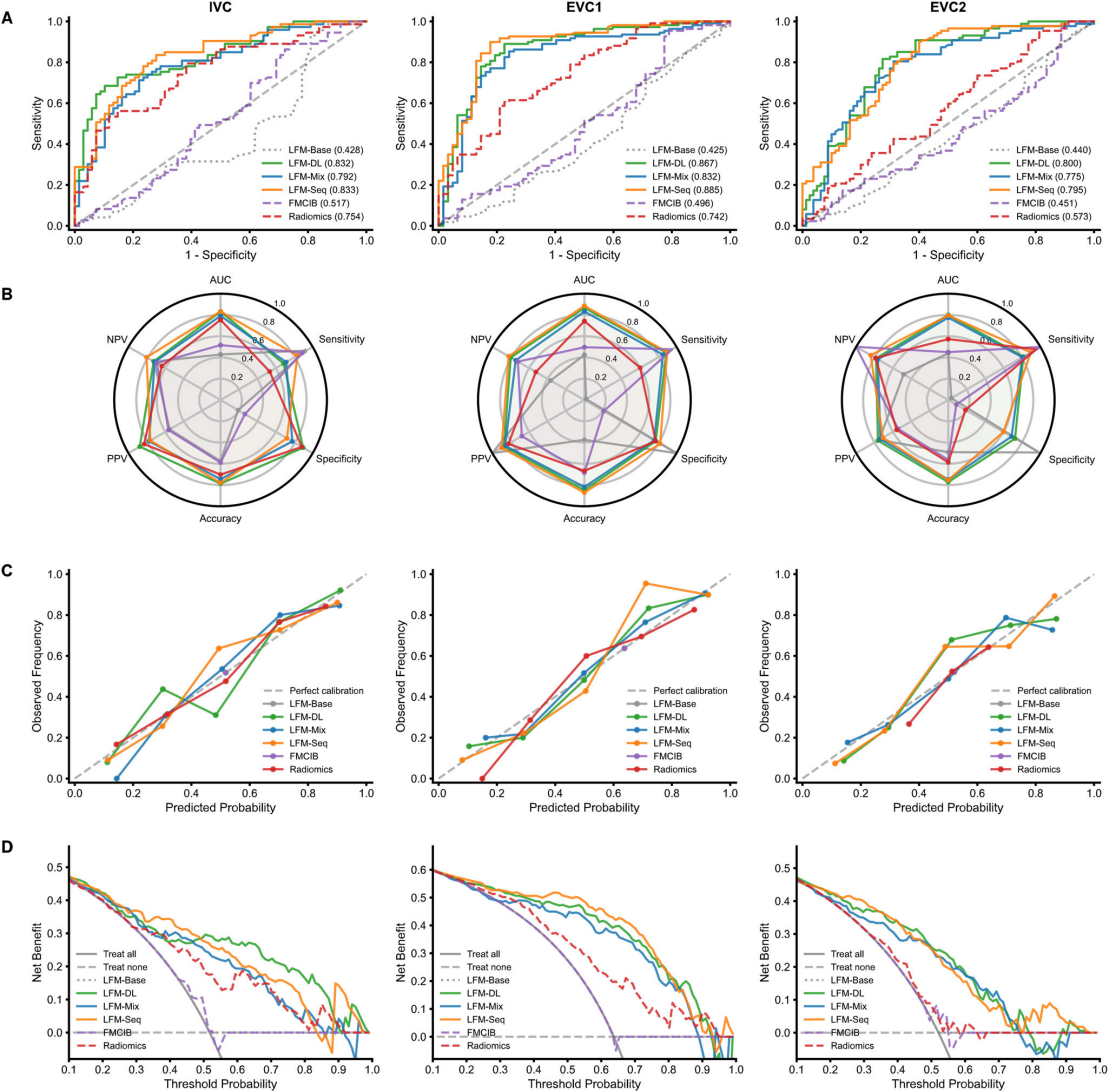
Performance of models for non‐invasive pathological grade assessment. (A) Receiver operating characteristic curves for pathological grade assessment in the internal validation cohort (IVC), external validation cohort 1 (EVC1), and external validation cohort 2 (EVC2). (B) Radar plots summarizing multidimensional assessment performance, including AUC, sensitivity, specificity, accuracy, positive predictive value (PPV), and negative predictive value (NPV). (C) Calibration curves comparing model‐assessed probabilities of high pathological grade with observed frequencies across the three cohorts. (D) Decision curve analysis showing the net benefit of each model across a range of threshold probabilities.

For non‐invasive assessment of MVI, LFM‐Seq achieved the best overall discrimination across the internal validation cohort and two external validation cohorts (Figure 2A). LFM‐Seq attained the highest AUC across all internal and external validation cohorts, reaching 0.916 (95% CI, 0.870–0.957) in the IVC and delivering consistent performance of 0.846 (95% CI, 0.784–0.900) in EVC1 and 0.920 (95% CI, 0.875–0.959) in EVC2. In comparison, LFM‐Base showed limited generalization, with AUCs of 0.596 and 0.640 in EVC1 and EVC2, whereas Radiomics and FMCIB yielded AUCs of 0.811 and 0.786 in EVC1, and 0.750 and 0.824 in EVC2, respectively. These findings indicate that lesion‐level CT pretraining, particularly sequential pretraining from generic lesions to HCC‐specific lesions, improved the transferability of imaging features for MVI assessment.

Beyond AUC, LFM‐Seq showed balanced assessment performance across multiple clinically relevant metrics (Figure 2B). It achieved sensitivities of 0.886, 0.960 and 0.979 and negative predictive values of 0.943, 0.957 and 0.964 in the IVC, EVC1 and EVC2, respectively. Specificities were 0.845, 0.698 and 0.740 across the three cohorts, with corresponding positive predictive values of 0.722, 0.713 and 0.829. Calibration curves showed generally acceptable agreement between model‐assessed and observed MVI probabilities (Figure 2C). Quantitative calibration metrics, including Brier scores, calibration intercepts, and calibration slopes, are provided in Table S15. The decision curve analysis demonstrated positive net benefit across a broad range of clinically relevant threshold probabilities, particularly for LFM‐derived models compared with Radiomics and FMCIB (Figure 2D).

For pathological grade assessment, LFM‐Seq achieved the highest AUC in the IVC and EVC1, with values of 0.833 (95% CI, 0.758–0.896) and 0.885 (95% CI, 0.826–0.935), respectively, and showed comparable performance to LFM‐DL in EVC2, with AUCs of 0.795 (95% CI, 0.724‐0.860) and 0.800 (95% CI, 0.732–0.867), respectively (Figure 3A and Table S2). In the IVC, LFM‐Seq outperformed LFM‐Base (AUC, 0.428; *p* < 0.001) and FMCIB (AUC, 0.517; *p* < 0.001). In EVC1, LFM‐Seq showed higher AUC than LFM‐Base (0.425; *p* < 0.001), FMCIB (0.496; *p* < 0.001), Radiomics (0.742; *p* = 0.002) and LFM‐Mix (0.832; *p* = 0.010). In EVC2, LFM‐Seq outperformed LFM‐Base (AUC, 0.440; p < 0.001), FMCIB (AUC, 0.451; *p* < 0.001) and Radiomics (AUC, 0.573; *p* < 0.001), with no significant difference compared with LFM‐DL (AUC, 0.800; *p* = 0.821) or LFM‐Mix (AUC, 0.775; *p* = 0.428).

LFM‐Seq demonstrated consistently high sensitivity across all cohorts (0.836–0.899), while maintaining moderate‐to‐high specificity (0.600–0.823). Positive and negative predictive values ranged from 0.709 to 0.899 and from 0.803 to 0.842, respectively (Figure 3B and Table S2). Calibration curves showed acceptable agreement between predicted and observed high‐grade probabilities (Figure 3C). Quantitative calibration metrics, including Brier scores, calibration intercepts, and calibration slopes, are provided in Table S16. The decision curve analysis suggested positive net benefit across clinically relevant thresholds (Figure 3D).

To dissect the contribution of each component of LFM‐Seq, we performed ablation experiments under otherwise identical training settings, including single‐phase inputs (arterial phase [AP]_only and portal venous‐phase [PV]_only), replacing cross‐phase attention with simple concatenation (simple_fusion), and replacing ABMIL with mean or max pooling (mean_pool and max_pool) (Tables S17 and S18). For MVI assessment, removing either imaging phase or the cross‐phase attention consistently reduced the AUC across all three cohorts, with the degradation reaching statistical significance in both external validation cohorts (EVC1: AP_only, *p* = 0.040; PV_only, *p* = 0.040; EVC2: AP_only, *p* = 0.014; PV_only, *p* = 0.005), and for simple_fusion in the IVC (*p* = 0.017). Replacing ABMIL with mean or max pooling also degraded performance, most notably in EVC2 (mean_pool, *p* = 0.002; max_pool, *p* < 0.001) and for max pooling in the IVC (*p* = 0.023). Similar trends were observed for pathological grade assessment (EVC1 AP_only, *p* < 0.001; max_pool, *p* = 0.043). Overall, LFM‐Seq achieved the best performance in 5 of the 6 cohort‐task combinations, and the advantage of the full model was more pronounced in the external validation cohorts, suggesting a trend toward improved cross‐center generalization with the full model.

### 2.3 Robustness of MVI and Pathological Grade Assessment

Robustness was evaluated under two clinically relevant conditions: tumor‐localization perturbation and limited training data (Figure 4). For tumor‐localization perturbation, bounding‐box coordinates of tumor regions were randomly perturbed from 0% to 25%, and model performance was evaluated across 50 repeated runs (Figure 4A and Tables S3 and S4). In MVI assessment, LFM‐Seq maintained AUCs of 0.840, 0.766 and 0.828 at the 25% perturbation level in the IVC, EVC1 and EVC2, respectively. These values were higher than those of LFM‐Base and FMCIB across all three cohorts, and were comparable to LFM‐Mix, which showed the smallest AUC decrease under perturbation. The AUC decreases of LFM‐Seq from 0% to 25% perturbation were 0.077, 0.080 and 0.091 across the three cohorts, smaller than those of LFM‐Base, LFM‐DL and FMCIB in all cohorts. For pathological grade assessment, LFM‐Seq maintained AUCs of 0.737, 0.799 and 0.715 at 25% perturbation, remaining higher than LFM‐Base and FMCIB across all three cohorts.

**FIGURE 4:**
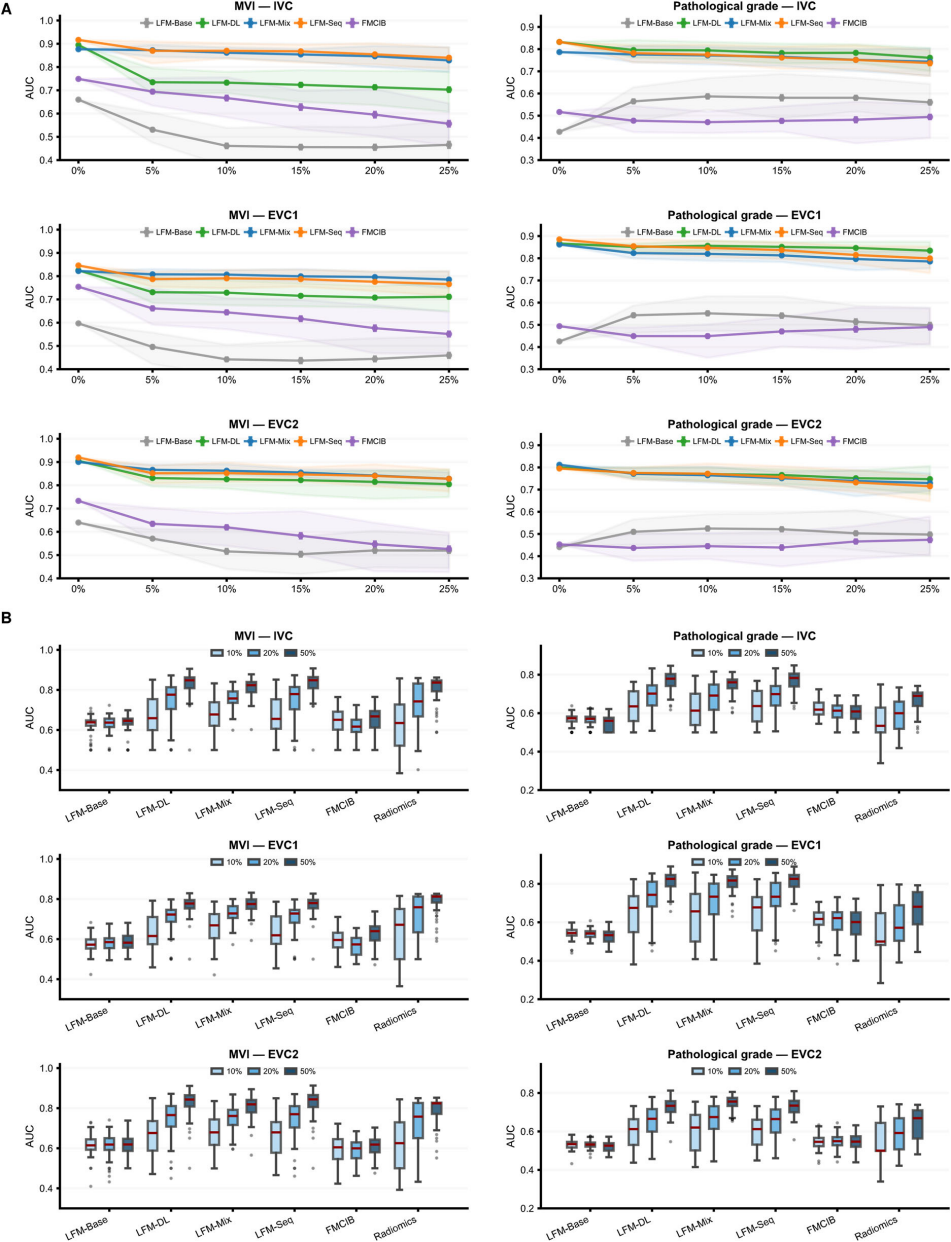
Robustness of MVI and pathological grade assessment under tumor‐localization perturbation and limited training data. (A) Model performance under tumor‐localization perturbation. Tumor bounding‐box coordinates were randomly perturbed from 0% to 25%, and AUCs were evaluated for MVI and pathological grade assessment in the internal validation cohort (IVC), external validation cohort 1 (EVC1), and external validation cohort 2 (EVC2). Lines indicate mean AUCs across repeated runs, and shaded areas indicate 95% confidence intervals. (B) Model performance under limited training data settings. Models were retrained using 10%, 20%, and 50% of the training cohort with stratified random sampling, and AUC distributions are shown for MVI and pathological grade assessment across the three cohorts.

Under limited training data settings, all models were retrained or fine‐tuned using 10%, 20% and 50% of the training cohort across 50 repeated stratified sampling runs (Figure 4B and Tables S5 and S6). Across both MVI and pathological grade assessment, CT‐pretrained LFM variants consistently outperformed LFM‐Base and FMCIB, particularly when the training set was reduced. LFM‐Seq showed clear advantages over LFM‐Base and FMCIB across all sampling proportions in every cohort, while maintaining performance comparable to LFM‐DL and LFM‐Mix. Notably, at the 50% training‐data setting, LFM‐Seq preserved high AUC levels for both MVI assessment and pathological grade assessment, whereas LFM‐Base and FMCIB remained substantially lower.

We further evaluated model performance across tumor size subgroups, including tumors ≤ 3 cm, 3–5 cm and > 5 cm (Figure S2 and Table S7). For MVI assessment, LFM‐Seq remained competitive across tumor size subgroups and cohorts. In tumors ≤ 3 cm, LFM‐Seq achieved AUCs of 1.000, 0.900 and 0.947 in the IVC, EVC1 and EVC2, respectively, exceeding LFM‐Base, FMCIB and Radiomics across all cohorts. In the 3–5 cm subgroup, LFM‐Seq also showed high AUCs across cohorts, with values of 0.871, 0.905 and 0.974, and remained higher than non‐LFM baselines. For tumors > 5 cm, LFM‐Seq achieved AUCs of 0.784, 0.769 and 0.844, maintaining higher overall performance than LFM‐Base and Radiomics, while showing comparable performance to LFM‐DL and FMCIB in some cohorts.

For pathological grade assessment, LFM‐Seq also maintained competitive performance across tumor size subgroups (Figure S3 and Table S8). In tumors ≤ 3 cm, LFM‐Seq achieved the highest AUC among all models in each cohort, yielding AUC values of 0.645, 0.793, and 0.908. In the 3–5 cm subgroup, LFM‐Seq achieved AUCs of 0.794, 0.890 and 0.800, showing the highest AUC in the IVC and EVC1 and comparable performance to LFM‐DL in EVC2. In tumors > 5 cm, LFM‐Seq achieved AUCs of 0.848, 0.747 and 0.674, remaining higher than LFM‐Base and FMCIB across cohorts.

### 2.4 RFS Prediction Using LFM‐Derived Whole‐Tumor Features

We further evaluated the prognostic value of LFM‐derived whole‐tumor features for RFS prediction. In the RFS development cohort and the independent validation cohort, LFM‐derived models showed higher or comparable C‐indexes than Clinical, Clinicopathological, Radiomics and FMCIB models (Figure 5A and Table S9). In EVC1, LFM‐Seq achieved the highest C‐index of 0.728 (95% CI, 0.674‐0.778), followed closely by LFM‐DL and LFM‐Mix, and significantly outperformed the Clinicopathological model (C‐index, 0.677, *p* = 0.029). In EVC2, LFM‐Seq achieved a C‐index of 0.701 (95% CI, 0.647‐0.755), which was significantly higher than Radiomics (C‐index, 0.636, *p* = 0.008) and FMCIB (C‐index, 0.625, *p* = 0.009).

**FIGURE 5:**
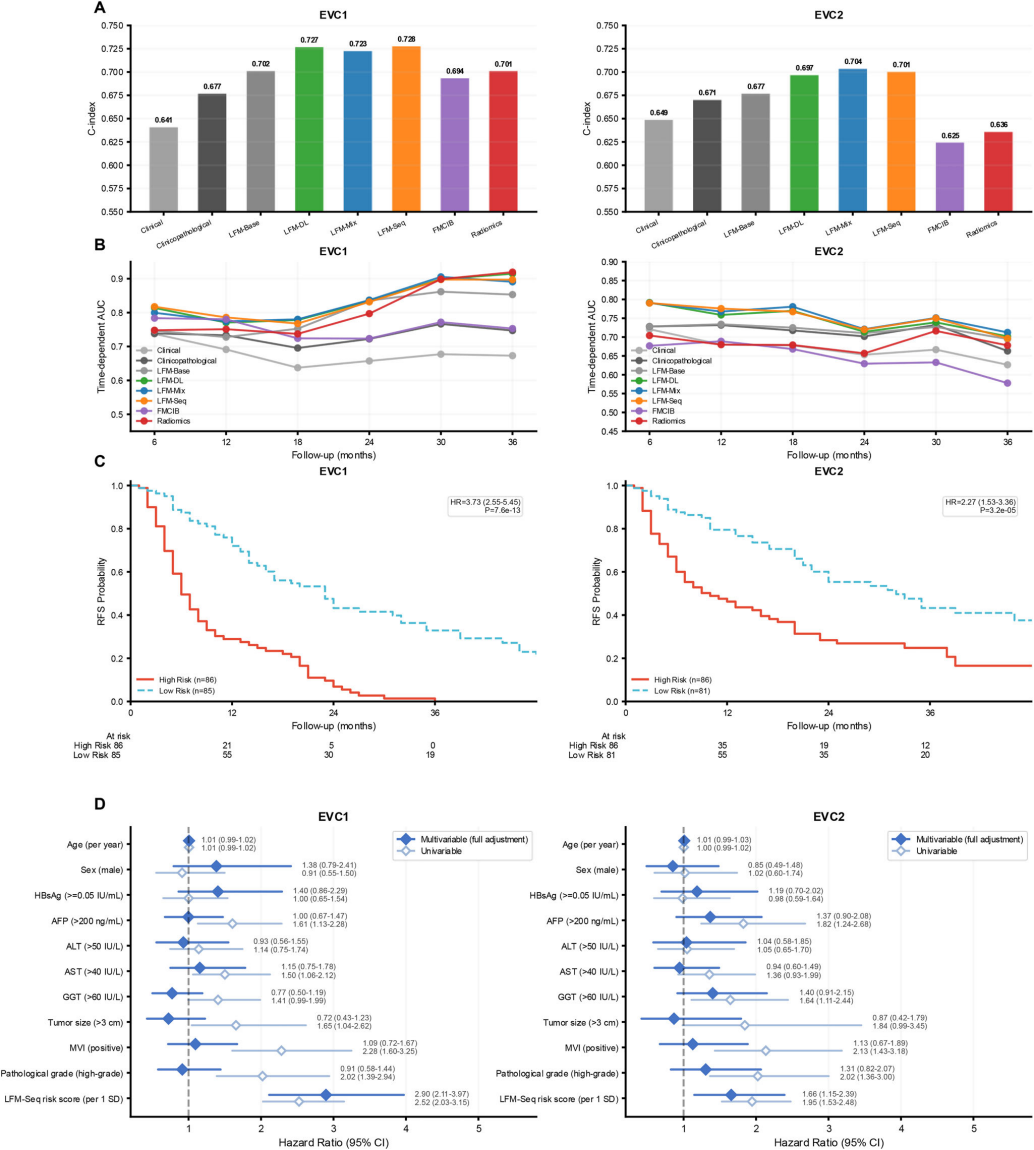
RFS prediction performance and prognostic value of models. (A) C‐index comparison of models for recurrence‐free survival (RFS) prediction in RFS development cohort (EVC1) and independent validation cohort (EVC2). (B) Time‐dependent AUC curves of different models for RFS prediction across follow‐up time points in EVC1 and EVC2. (C) Kaplan‐Meier curves showing RFS stratification between high‐ and low‐risk groups defined by the LFM‐Seq‐derived risk score in EVC1 and EVC2. Hazard ratios (HRs) and log‐rank *p* values are shown. (D) Univariable and multivariable Cox regression analyses evaluating the association between features and RFS. Diamonds indicate hazard ratios. The LFM‐Seq risk score is expressed per 1‐standard‐deviation increment, using EVC1 as the reference for standardization.

Time‐dependent ROC analysis further showed consistent RFS prediction performance of LFM‐Seq across follow‐up time points (Figure 5B and Table S9). In EVC1, LFM‐Seq achieved 12‐, 24‐ and 36‐month AUCs of 0.786, 0.832 and 0.897, respectively. It significantly outperformed the Clinicopathological model at 24 months (*p* = 0.001) and 36 months (*p* < 0.001), and outperformed FMCIB at 24 months (*p* = 0.012) and 36 months (*p* = 0.019). In EVC2, LFM‐Seq achieved 12‐, 24‐ and 36‐month AUCs of 0.776, 0.720 and 0.696, respectively, and significantly outperformed FMCIB across all three time points (*p* = 0.029, *p* = 0.045 and *p* = 0.028, respectively). Compared with Radiomics, LFM‐Seq showed higher 12‐month AUC in EVC2 (*p* = 0.004).

Kaplan–Meier analysis based on LFM‐Seq risk scores showed separation between high‐ and low‐risk groups in the external cohorts (Figure 5C). In Cox regression analyses performed separately in each external cohort, the LFM‐Seq risk score (per 1 SD increment, with EVC1 as the reference for standardization) was significantly associated with RFS in univariable analysis (EVC1: hazard ratio (HR), 2.525 [95% CI, 2.026–3.146]; EVC2: HR, 1.945 [1.527–2.477]; both *p* < 0.001) and remained independently associated with RFS after full adjustment for all clinicopathological variables (EVC1: HR, 2.896 [2.112–3.971], *p* < 0.001; EVC2: HR, 1.657 [1.148–2.392], *p* = 0.007; Figure 5D, Table S10).

The proportional‐hazards assumption was satisfied for the LFM‐Seq risk score in EVC1 (Schoenfeld *p* = 0.950) but was violated in EVC2 (*p* = 0.013). Time‐dependent HRs showed mild attenuation over time in EVC1 (HR, 2.68 at 6 months to 2.03 at 36 months; time‐interaction *p* = 0.234), whereas the effect attenuated markedly in EVC2 (HR, 2.56 at 6 months to 1.00 at 36 months; time‐interaction *p* < 0.001; Table S13). These findings suggest that the risk score may reflect early‐recurrence risk, as 86.3% of recurrences in EVC1 and 84.9% in EVC2 occurred within 24 months after resection. In addition, a sensitivity analysis was performed using a stratified Cox model in which AFP status and age (dichotomized at the cohort median) were handled as stratification factors with stratum‐specific baseline hazards, and the adjusted association of the LFM‐Seq risk score remained essentially unchanged (EVC1: HR 3.063 [2.201–4.263], *p* < 0.001; EVC2: HR 1.684 [1.151–2.462], *p* = 0.007).

Incremental value analysis using nested models (a clinical model with versus without the LFM‐Seq risk score) showed that adding the LFM‐Seq risk score significantly improved risk discrimination and reclassification in both EVC1 (IDI, 0.162–0.221; continuous NRI, 0.303–0.584; all *P* ≤ 0.004 across 6–36 months) and in EVC2 at 6, 12, and 24 months for both IDI and NRI (IDI, 0.073–0.092, *P* ≤ 0.016; continuous NRI, 0.244–0.342, *P* ≤ 0.024), with the IDI remaining significant at 36 months (0.074, *p* = 0.020) and a borderline continuous NRI (0.197, *p* = 0.064). The detailed IDI and NRI values with 95% CI were listed in Table S14.

In addition, we also evaluated whether the LFM‐Seq risk score provided incremental prognostic value beyond the Clinicopathological model, which additionally incorporated MVI and pathological grade. In EVC1, adding the LFM‐Seq risk score significantly improved discrimination and reclassification at all evaluated time points from 6 to 36 months (IDI, 0.139–0.169, all *p* < 0.001; continuous NRI, 0.357–0.602, all *P* ≤ 0.004). Importantly, this incremental value was also observed in the independent validation cohort EVC2 at 6, 12, and 24 months (IDI, 0.040–0.052, *p* = 0.024–0.040; continuous NRI, 0.220–0.269, *p* = 0.032), whereas both measures were borderline at 36 months (IDI, 0.039, *p* = 0.052; continuous NRI, 0.213, *p* = 0.052). Detailed results are provided in Table S19.

We further performed tumor size subgroup analysis for RFS prediction in patients with tumors ≤ 3 cm, 3–5 cm and > 5 cm (Figures S4 and S5 and Tables S11 and S12). Across the two cohorts, LFM‐Seq remained among the top‐performing models across tumor size strata. For C‐index, LFM‐Seq achieved values of 0.658‐0.751 in tumors ≤ 3 cm, 0.680‐0.732 in tumors 3–5 cm and 0.649‐0.689 in tumors > 5 cm. Compared with the Clinicopathological model, LFM‐Seq showed higher C‐indexes across all tumor size subgroups in both cohorts, and it also exceeded FMCIB in most subgroup comparisons. Time‐dependent AUC analysis showed similar trends, with LFM‐Seq maintaining competitive 12‐, 24‐ and 36‐month RFS prediction performance across tumor size strata, particularly in small and intermediate‐sized tumors. In larger tumors, subgroup performance was more variable across models, although LFM‐derived models generally remained comparable to or higher than clinical, Radiomics and FMCIB models.

### 2.5 Visual Plausibility and Biological Interpretation of LFM‐Derived Imaging Phenotypes

To assess whether LFM‐derived imaging phenotypes were visually plausible, we generated positive‐class occlusion heatmaps to localize image regions contributing to MVI‐positive and high‐grade outputs. For each task, contribution was defined as the decrease in positive‐class probability following local occlusion. In the aggressive case, positive‐class contribution regions were more prominent and were primarily distributed within and adjacent to the tumor boundary, whereas the non‐aggressive case showed less conspicuous contribution patterns (Figure 6). The heatmaps also suggested task‐dependent patterns, with MVI‐positive contribution appearing relatively focal and high‐grade contribution showing more diffuse involvement.

**FIGURE 6:**
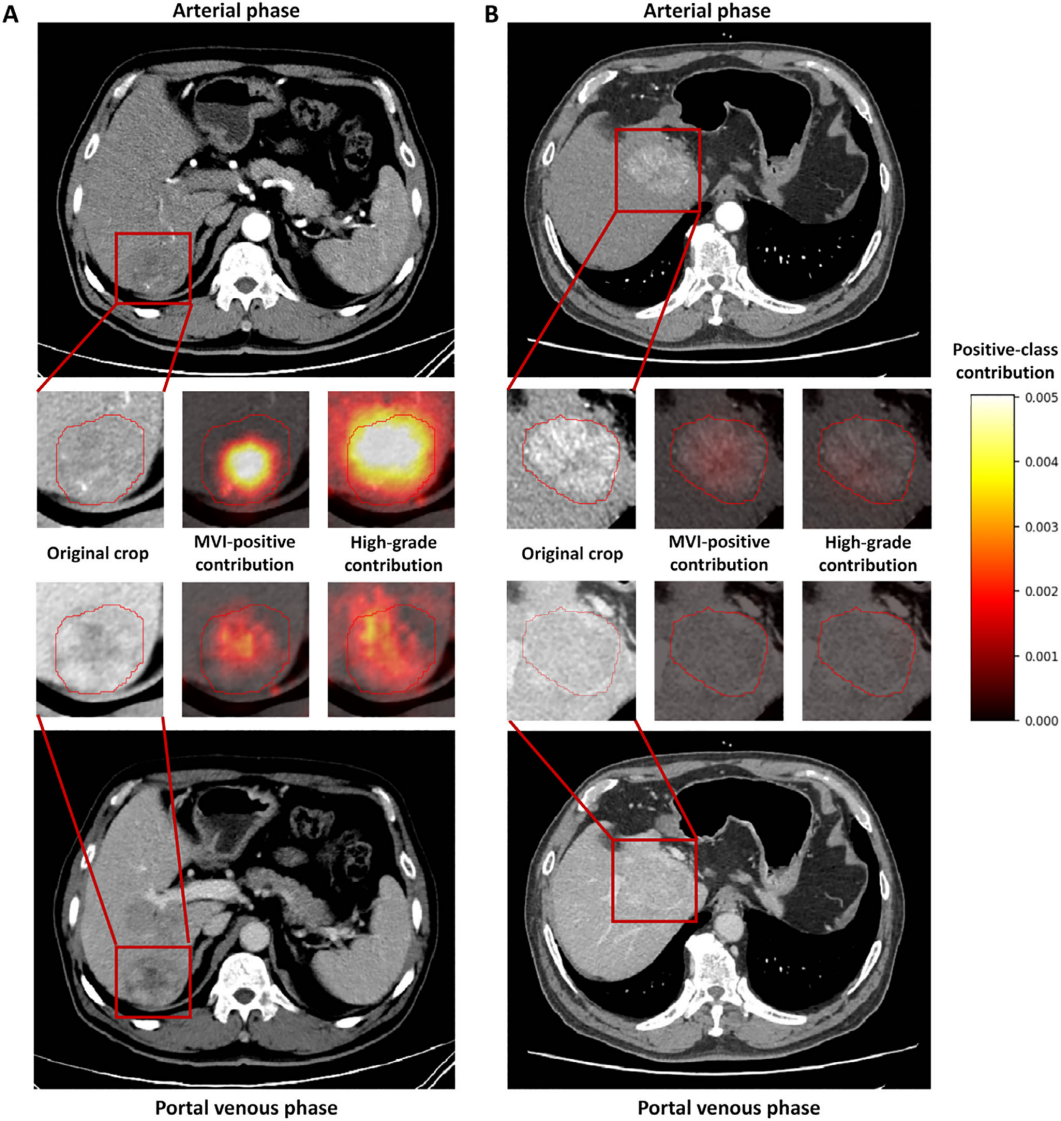
Visual interpretation of LFM‐based MVI and pathological grade assessment. Representative cases showing positive‐class occlusion heatmaps for MVI and pathological grade on arterial‐phase and portal venous‐phase CT images. (A) A representative HCC case with MVI‐positive and high pathological grade. (B) A representative HCC case with MVI‐negative and low pathological grade. For each case, magnified tumor crops are shown with corresponding heatmaps for MVI‐positive and high‐grade contribution. Red boxes indicate tumor locations, and red contours denote tumor boundaries. Positive‐class contribution was defined as the decrease in MVI‐positive or high‐grade probability after local occlusion. Warmer colors indicate regions with higher contribution to MVI‐positive or high‐grade probability.

To quantitatively characterize the specific imaging phenotypes captured by LFM‐Seq, we further measured the overlap between high‐contribution regions and the tumor and peritumoral regions (Tables S20 and S21). For MVI‐positive patients, AP high‐contribution regions were predominantly localized within the tumor, with a median in‐tumor overlap of 100.0% across the three cohorts. In contrast, the high‐contribution regions of PV phase had a median in‐tumor overlap of 36.2% and increased to 74.7% after inclusion of the 10‐pixel peritumoral region. AP overlap was significantly greater than PV overlap within patients (median paired difference, 35.7%; Wilcoxon signed‐rank *p* = 6 × 10^−^^7^). For high pathological grade, high‐contribution regions showed broader distributions in both phases, with median in‐tumor overlaps of 67.4% for AP and 61.8% for PV. The AP–PV difference was smaller than that observed for MVI (median paired difference, 4.1%; *p* = 0.021). Some examples were also displayed in Figure S6. These findings demonstrate task‐ and phase‐dependent spatial patterns in the imaging phenotypes captured by LFM‐Seq.

To explore the biological relevance of the LFM‐derived recurrence risk phenotype, we linked LFM‐Seq risk stratification with matched transcriptomic data in the TCGA‐LIHC cohort. The imaging‐genomic cohort included 35 patients, comprising 22 patients in the LFM‐Seq‐derived high‐risk group and 13 patients in the low‐risk group. Differential expression analysis identified 126 differentially expressed genes (DEGs) between the two groups using an FDR < 0.10 and an absolute log2 fold change > 0.5, including 60 genes upregulated and 66 genes downregulated in the high‐risk group (Figure 7A).

**FIGURE 7:**
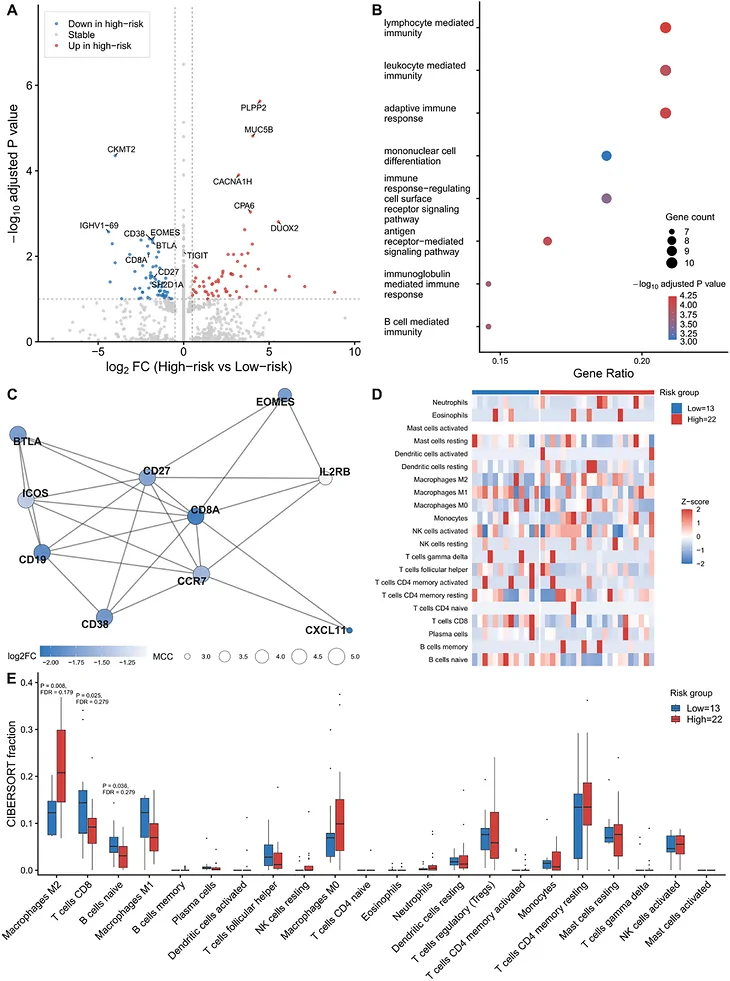
Exploratory radiogenomic associations of the LFM‐Seq risk score. (A) Volcano plot showing differentially expressed genes (DEGs) between the LFM‐Seq‐derived high‐risk (*n* = 22) and low‐risk (*n* = 13) groups. Using a Benjamini–Hochberg false discovery rate (FDR) < 0.10 and an absolute log2 fold change > 0.5 as the significance thresholds, 60 genes were upregulated and 66 genes were downregulated in the high‐risk group. Adjusted *p* values were calculated using the Benjamini–Hochberg procedure. (B) Gene Ontology biological process enrichment analysis of genes downregulated in the high‐risk group, with Benjamini–Hochberg FDR correction for multiple testing. (C) Protein–protein interaction network of top hub genes downregulated in the high‐risk group. (D) Heatmap of CIBERSORT‐estimated immune‐cell fractions across the LFM‐Seq‐derived low‐risk (*n* = 13) and high‐risk (*n* = 22) groups. Colors indicate row‐wise z‐score‐normalized immune‐cell fractions. (E) Boxplots comparing CIBERSORT‐estimated immune‐cell fractions between low‐risk (*n* = 13) and high‐risk (*n* = 22) groups. Comparisons were performed using two‐sided Wilcoxon rank‐sum tests, followed by Benjamini–Hochberg FDR correction across the 22 immune‐cell types. The nominal and FDR adjusted *p* values were shown in the image.

Gene Ontology biological process enrichment analysis was then performed separately for genes downregulated and upregulated in the high‐risk group. Genes downregulated in the high‐risk group were predominantly enriched in immune‐related processes, including lymphocyte‐mediated immunity, leukocyte‐mediated immunity, adaptive immune response, antigen receptor‐mediated signaling pathway and B‐cell‐mediated immunity (Figure 7B), whereas no significant Gene Ontology biological process terms were identified for genes upregulated in the high‐risk group.

To further characterize the immune‐related molecular network underlying these transcriptional differences, we constructed a protein‐protein interaction network using genes downregulated in the high‐risk group. The network identified several immune‐related hub genes, including CD8A, CD27, CD38, EOMES, BTLA, CCR7 and IL2RB, which are involved in T‐cell activation, B‐cell signaling, chemokine‐mediated trafficking and immune regulation (Figure 7C).

CIBERSORT immune‐cell deconvolution was further performed to explore potential associations between the LFM‐derived risk phenotype and immune‐cell composition (Figure 7D,E). In uncorrected two‐sided Wilcoxon rank‐sum tests, the high‐risk group showed a nominally higher M2 macrophage fraction (median, 0.208 vs. 0.129; *p* = 0.008) and nominally lower CD8 T‐cell (median, 0.092 vs. 0.144; *p* = 0.025) and naive B‐cell (median, 0.031 vs. 0.051; *p* = 0.036) fractions than the low‐risk group. However, none of these differences remained statistically significant after Benjamini–Hochberg FDR correction across the 22 immune‐cell types (FDR = 0.179, 0.279, and 0.279, respectively). Given the limited sample size of the imaging‐genomic cohort, these immune‐cell findings should be interpreted as exploratory associations.

## 3 Discussion

In this multicenter study, we developed and systematically evaluated a CT lesion foundation model framework for non‐invasive assessment of HCC aggressiveness and recurrence risk. Our central finding is that lesion‐level CT self‐supervised pretraining, particularly sequential pretraining from generic lesions to HCC‐specific lesions, improved performance across pathological aggressiveness assessment and postoperative RFS prediction. LFM‐Seq achieved externally validated performance for MVI and pathological grade assessment, retained robustness under tumor localization perturbation and limited training data, and provided independent prognostic information for RFS beyond established clinicopathological variables. In addition, visual interpretation and genomic analyses provided exploratory support for the spatial and biological plausibility of LFM‐Seq‐derived imaging phenotypes. Collectively, these findings suggest that LFM‐Seq captures intra‐lesional CT phenotypes that are relevant to pathological aggressiveness, informative for recurrence risk stratification, and interpretable at both imaging and genomic levels.

The clinical motivation for integrating MVI, pathological grade and RFS within a single framework is that they represent distinct yet interconnected manifestations of HCC aggressiveness. MVI and high pathological grade provide histopathological evidence of invasive behavior and poor differentiation, whereas postoperative recurrence reflects the clinical consequence of aggressive tumor biology following curative‐intent treatment [2, 3, 4, 5, 6, 7]. Current clinical staging and treatment algorithms are primarily based on tumor burden, liver function and performance status [2, 3], however, they may not fully reflect the biological aggressiveness that drives recurrence. Consequently, patients within the same clinical stage may still exhibit markedly different recurrence risks. The ability of LFM‐derived features to assess MVI and pathological grade while simultaneously predicting RFS suggests that lesion‐level CT phenotypes could capture a broader aggressive tumor state, supporting their use as unified imaging biomarkers for pathological characterization and recurrence risk stratification in HCC.

The comparison among LFM variants and previous baseline models offers insight into feature learning for lesion CT images. The LFM‐Base model, initialized from a general visual foundation model without lesion‐level CT adaptation, underperformed CT‐pretrained LFM variants, especially for pathological grade assessment. This is consistent with the substantial domain gap between natural images and CT images, where clinically relevant signals are expressed as soft‐tissue attenuation, enhancement patterns, and subtle textural heterogeneity rather than object‐centric visual semantics [23, 28]. LFM‐DL markedly improved over LFM‐Base, indicating that generic lesion CT pretraining can provide transferable features for HCC characterization. LFM‐Seq further improved performance, suggesting that a sequential curriculum from broad lesion phenotypes to HCC‐specific lesions may better preserve general CT lesion knowledge while adapting features to HCC‐specific aggressive patterns. Furthermore, pooled joint pretraining (LFM‐Mix) yielded no improvement over generic lesion pretraining alone and even degraded MVI performance in the external cohorts, consistent with dilution of general lesion knowledge, whereas sequential adaptation recovered and exceeded both, suggesting that the sequential curriculum acts as a regularizer that preserves generic lesion features while aligning them with HCC‐specific patterns. Importantly, LFM‐Seq also outperformed Radiomics and FMCIB [21] in most MVI and pathological grade comparisons. Radiomics relies on predefined handcrafted features and is sensitive to segmentation, preprocessing, and feature‐selection procedures [13, 14, 17], whereas FMCIB represents a 3D foundation model that may capture volumetric context but is not specifically optimized for lesion‐centric, multiphase HCC images [21]. By contrast, the integration of LFM and ABMIL enables adaptive aggregation of 2D lesion patch‐level features across variable‐length tumor slices, while preserving the capacity to model heterogeneous intratumoral regions. This design is consistent with recent studies showing the value of multichannel and patch‐level feature fusion for tumor prediction modelling [29, 30]. The ablation experiments further validated the architectural design of LFM‐Seq. Single‐phase inputs consistently underperformed the dual‐phase model, confirming that arterial and portal venous phases provide complementary enhancement information for characterizing HCC aggressiveness. Replacing cross‐phase attention with simple concatenation significantly degraded performance, indicating that explicit bidirectional feature interaction between phases, rather than naive feature stacking, underlies the benefit of multiphase input. Similarly, the superiority of ABMIL over mean and max pooling supports the value of attention‐weighted aggregation in identifying discriminative tumor regions from variable‐length slice sequences. Notably, the performance degradation caused by removing these components was more pronounced in the external validation cohorts, suggesting that these components may contribute to cross‐center generalizability.

The robustness analyses further highlight the advantage of LFM‐Seq over baseline models under clinically realistic constraints. In practice, tumor localization and bounding‐box placement can vary across radiologists, whereas precise 3D segmentation is labor‐intensive. Radiomics is particularly vulnerable to such variability because many handcrafted features are directly determined by precise tumor boundary definition [13, 14, 17]. Although FMCIB can extract 3D imaging features without requiring precise tumor segmentation, it showed greater performance degradation under tumor localization perturbation. By contrast, LFM‐Seq maintained more stable and consistently higher performance, suggesting that its predictions were less dependent on fragile boundary‐level cues. Furthermore, the limited‐data experiments further demonstrated the data efficiency of LFM‐Seq. When the training set was reduced, CT‐pretrained LFM variants consistently outperformed LFM‐Base and FMCIB, while LFM‐Seq maintained performance comparable to or higher than other CT‐pretrained variants in most settings. This suggests it learns transferable features that reduce dependence on large task‐specific annotated datasets. This is particularly relevant for HCC research, as multiphase CT datasets with surgical pathology and recurrence follow‐up are difficult to assemble [13, 14, 18, 21].

Building on the pathology‐informed whole‐tumor features learned during MVI and pathological grade assessment, we further evaluated their prognostic value for RFS prediction. LFM‐Seq achieved competitive C‐index and time‐dependent AUC values in external cohorts. Although these whole‐tumor features were initially learned under MVI and pathological grade supervision, the LFM‐Seq risk score remained independently associated with RFS after full adjustment for these pathological variables and other clinicopathological factors. Moreover, adding the LFM‐Seq risk score to a clinicopathological model that already incorporated MVI and pathological grade further improved risk discrimination and reclassification. This suggests that the learned representation is not merely a surrogate for MVI or pathological grade and appears to carry additional prognostic information beyond these pathological labels. Notably, the prognostic effect of the LFM‐Seq risk score was strongest for early recurrence and progressively attenuated over time. One biologically plausible interpretation of this temporal pattern is that early recurrence after hepatic resection for HCC largely reflects occult intrahepatic metastasis from the primary tumor, driven by its intrinsic aggressiveness—characterized by MVI and poor differentiation [5]—phenotypes that are captured by the LFM‐Seq imaging signature. In contrast, late recurrence more commonly represents de novo hepatocarcinogenesis arising from the underlying diseased liver parenchyma. Consistent with this distinction, 86.3% and 84.9% of recurrences in EVC1 and EVC2, respectively, occurred within the first two years after resection. Clinically, these findings indicate that LFM‐Seq‐based risk stratification is most informative during the early postoperative period, when intensified surveillance and earlier therapeutic intervention may confer the greatest benefit.

Incremental value analyses using IDI and NRI further showed added risk reclassification over the clinical model. Prior studies have investigated radiomics or deep learning for HCC recurrence prediction from preoperative images [13, 14, 15, 16]. Our approach differs by using pathological aggressiveness assessment to learn patient‐level lesion features and then extending these features to recurrence risk stratification, thereby linking histopathological characterization with prognostic modeling.

The tumor size subgroup analyses provide a clinically relevant perspective on the potential utility of LFM‐derived phenotypes. Tumor size is central to HCC staging and treatment selection, but it does not fully reflect tumor biology. In our study, LFM‐Seq maintained competitive performance across various tumor size subgroups for both aggressiveness assessment and RFS prediction. This is particularly relevant for small HCCs, in which patients are often considered candidates for curative treatment but may still harbor MVI, poor differentiation, or early recurrence risk. The preserved performance in small and intermediate‐sized tumors suggests that LFM‐Seq may help identify biologically aggressive tumors that are not adequately characterized by size alone. In larger tumors, model performance was more variable, which may reflect greater intratumoral heterogeneity, necrosis and heterogeneous enhancement patterns. Importantly, the LFM‐Seq risk score remained independently associated with RFS after adjustment for tumor size and pathological factors, supporting that the model captured prognostic information beyond tumor burden.

The visual interpretation analyses further support the plausibility of LFM‐Seq. Quantitative region‐level attribution revealed distinct task‐ and phase‐dependent spatial patterns. For MVI assessment, AP high‐contribution regions were predominantly localized within the tumor, whereas PV contributions were more broadly distributed toward the tumor–peritumoral interface. This phase‐dependent pattern may reflect complementary enhancement information related to tumor vascularity and invasive behavior: arterial‐phase images may emphasize heterogeneous intratumoral perfusion and enhancement, whereas portal venous‐phase images may better capture differences extending toward the tumor margin and adjacent parenchyma. In contrast, pathological grade showed a broader lesion‐ and boundary‐associated distribution in both phases, which may be compatible with the more global nature of tumor differentiation and intratumoral heterogeneity rather than a single focal invasive feature. These findings suggest that different manifestations of HCC aggressiveness are associated with distinct spatial imaging patterns within the learned LFM representation.

Beyond visual plausibility, exploratory genomic analyses provided exploratory but biologically plausible associations with the LFM‐Seq risk score. At the molecular level, the LFM‐derived high‐risk group showed coordinated downregulation of immune‐related genes and pathways involving lymphocyte‐mediated immunity, adaptive immune response, B‐cell‐mediated immunity, and immune receptor signaling. At the cellular level, the high‐risk group was accompanied by nominally reduced CD8 T‐cell and naive B‐cell fractions and increased M2 macrophage fraction [31, 32, 33]. These findings are consistent with recent HCC imaging studies linking non‐invasive imaging biomarkers to tumor heterogeneity and immune microenvironment phenotypes [34, 35]. However, none of these immune‐cell differences remained statistically significant after FDR correction. Therefore, the immune‐cell findings should be regarded as exploratory and hypothesis‐generating associations rather than evidence of a definitive immune‐microenvironmental mechanism. These preliminary radiogenomic associations warrant validation in larger independent imaging‐genomic cohorts.

The intended clinical scenario for LFM‐Seq is preoperative risk stratification in patients with resectable HCC undergoing routine CECT before curative‐intent surgery. Because MVI and pathological grade are generally established only after resection, non‐invasive assessment of these aggressive pathological features from preoperative CT could provide complementary information for multidisciplinary treatment planning. In particular, previous studies have suggested that preoperative estimation of MVI risk may assist surgical planning, including consideration of anatomical versus non‐anatomical resection [36, 37]. In addition, the LFM‐derived recurrence‐risk score may help identify patients who warrant closer postoperative surveillance, particularly during the early postoperative period [2, 3, 6, 7].

Several limitations should be acknowledged. First, although this study included three independent centers and external validation cohorts, the supervised cohorts were retrospectively collected and showed substantial differences in demographic and clinicopathological characteristics. Such differences in case mix and disease severity may contribute to variation in model discrimination and calibration across centers. In addition, CT examinations were acquired using heterogeneous scanner platforms and acquisition protocols. The independent effects of scanner model, acquisition parameters, and reconstruction settings could not be fully disentangled. Prospective validation in more diverse populations and scanner‐ or protocol‐stratified analyses is warranted to further assess model generalizability. Second, conventional radiologist‐defined semantic CT features were not systematically and uniformly recorded across the retrospective multicenter cohorts. Future prospective studies using standardized semantic imaging assessment are warranted to directly compare LFM‐Seq with conventional radiological risk stratification approaches. Third, because pathological labels were derived from the original institutional pathology reports without centralized pathological re‐review, potential interobserver and inter‐institutional variability in pathological assessment cannot be completely excluded. Fourth, the current LFM pipeline still requires tumor localization before feature extraction. Although tumor localization perturbation experiments demonstrated robustness to moderate annotation variability, future integration with reliable automated lesion detection or segmentation methods could further improve workflow efficiency and reproducibility. Fifth, the genomic analysis was based on a relatively small dataset with matched CT imaging and transcriptomic data. Therefore, these findings should be interpreted as exploratory associations and warrant validation in larger imaging‐genomic cohorts. Finally, although LFM‐Seq showed the most favorable calibration among the compared models, calibration intercepts deviated from 0 in the external cohorts, most prominently for MVI assessment in EVC2, consistent with between‐center differences in MVI prevalence. Center‐specific recalibration of predicted probabilities may therefore be needed before local clinical application. Moving forward, these avenues will be key to refining our methodology and improving the overall clinical impact of LFM‐Seq in individualized HCC risk stratification and treatment planning.

In conclusion, we developed a CT lesion foundation model LFM‐Seq for non‐invasive assessment of HCC aggressiveness and recurrence risk. By combining lesion‐level self‐supervised pretraining with multiple instance learning‐based whole‐tumor aggregation, LFM‐Seq captured intra‐lesional CT phenotypes associated with MVI, pathological grade, and recurrence risk. These findings support the potential of LFM‐Seq as a robust and interpretable imaging biomarker for individualized HCC risk stratification and treatment planning.

## 4 Methods

### 4.1 Study Population and Data Collection

Ethics approval for this study was obtained from the Institutional Review Board (RJ‐2024‐142). Due to the study's retrospective nature, the requirement for informed consent was waived. The data inclusion and exclusion flow diagram of this study is illustrated in Figure S1.

For construction of the pretraining datasets for LFM development, CT lesion patches were curated from four data sources. A generic lesion pretraining set was first constructed from DeepLesion [28], which included 4,427 patients with annotated lesions on CT. To further adapt the model to HCC‐specific imaging characteristics, an HCC‐specific pretraining set was constructed from three datasets with annotated HCC lesions on CT, including PLC [38], WAW [39] and MDA [40]. These datasets included 94, 233 and 105 patients, respectively.

For HCC aggressiveness assessment, patients with surgically resected HCC were retrospectively collected from three centers. In Center 1, 543 consecutive patients with HCC who underwent hepatectomy between February 2015 and June 2024 were initially screened. The inclusion criteria were: (1) pathologically confirmed HCC; (2) available clinical and pathological data; and (3) dynamic contrast‐enhanced abdominal CT performed within 4 weeks before hepatectomy. The exclusion criteria were: (1) preoperative antitumor treatment; (2) missing arterial‐phase or portal venous‐phase CT images; (3) suboptimal image quality or severe image artifacts; and (4) unsuccessful image registration. After exclusion of 139 patients, 404 patients from Center 1 were included and chronologically divided into a training cohort (TC, *n* = 263) and an internal validation cohort (IVC, *n* = 141).

Pathological information used as reference standards for HCC aggressiveness assessment was retrospectively collected from the final postoperative pathology reports recorded in the hospital information systems of the participating centers. MVI was defined as the presence of tumor cells or tumor thrombi within a vascular lumen lined by endothelial cells, occurring in portal vein branches, hepatic vein branches, or hepatic sinusoids within the tumor or in the adjacent liver parenchyma, and identifiable on microscopic examination of hematoxylin–eosin‐stained sections. MVI status was extracted as a binary pathological variable (present or absent) according to the original pathology reports. Pathological grade was recorded according to the Edmondson–Steiner grading system, which stratifies HCC into grades 1–4 based on the degree of histological differentiation, with higher grades indicating poorer differentiation, and grades were dichotomized as low‐grade HCC (grades 1–2) and high‐grade HCC (grades 3–4).

Two independent external validation cohorts were collected from Centers 2 and 3 using the same inclusion and exclusion criteria. In Center 2, 204 patients with HCC who underwent hepatectomy between September 2015 and July 2023 were initially screened. After exclusion of 33 patients, 171 patients were included as external validation cohort 1 (EVC1). In Center 3, 197 patients with HCC who underwent hepatectomy between January 2019 and May 2024 were initially screened. After exclusion of 30 patients, 167 patients were included as external validation cohort 2 (EVC2). The TC was used for supervised model training, whereas the IVC, EVC1 and EVC2 were used to evaluate model performance for MVI and pathological grade assessment.

For postoperative recurrence prediction, EVC1 was used as the RFS prediction model development cohort and EVC2 was used for independent model validation. Patients were followed every 3 months during the first 2 years after surgery and every 6 months thereafter, using serum alpha‐fetoprotein measurement and contrast‐enhanced imaging. The last follow‐up dates were August 2024 for EVC1 and December 2024 for EVC2. RFS was defined as the interval from surgery to the first occurrence of tumor recurrence or death from any cause, whichever occurred first. Death without documented recurrence was therefore treated as an RFS event. Patients who remained alive and recurrence‐free were censored at the date of their last disease assessment.

For biological interpretation, transcriptomic data from the Cancer Genome Atlas Liver Hepatocellular Carcinoma (TCGA‐LIHC) cohort [41] and matched CT imaging records from The Cancer Imaging Archive (TCIA) [42] were used to explore the biological basis of LFM‐derived risk scores. Ninety‐seven patients with available imaging records were initially identified. Patients were excluded because of missing preoperative CT, missing arterial‐phase or portal venous‐phase CT images, missing RNA‐sequencing data, or multiple subfoci/intrahepatic metastases. After exclusion of 62 patients, 35 patients with matched multiphase CT images and transcriptomic data were included as the biological analysis cohort.

### 4.2 CT Image Preprocessing

For the pretraining datasets, generic lesion patches were extracted from DeepLesion according to the available CT bounding‐box annotations, resulting in 32 120 generic lesion CT patches. Then, 6 387 HCC‐specific lesion patches were extracted from arterial‐phase or portal venous‐phase CT images according to the available lesion annotations from the PLC, WAW and MDA datasets. To reduce redundancy across adjacent slices, the slice with the largest tumor area was selected, and additional tumor‐containing slices were sampled at a five‐slice interval cranially and caudally within the tumor extent, resulting in 6 387 HCC‐specific CT patches. All pretraining patches were windowed using a soft‐tissue setting (width of 400 HU and level of 40 HU) and resized to 128 × 128 pixels.

For the supervised cohorts from Centers 1–3, arterial‐phase and portal venous‐phase CT images were spatially aligned before lesion extraction. Multiphase CT images of each patient were registered using a deformable registration pipeline implemented with the Insight Segmentation and Registration Toolkit (ITK, version 5.4.0), including rigid, affine and B‐spline transformation steps. Registration quality was visually reviewed, and cases with unsuccessful registration were excluded. Tumor regions were annotated by two radiologists with 15 years of experience in abdominal CT interpretation. One radiologist performed the initial tumor annotation, and the second radiologist reviewed the annotations. Both radiologists were blinded to postoperative pathological outcomes. Any discrepancies in tumor annotation were resolved through discussion and consensus between the two radiologists.

For each tumor‐containing slice, a square region enclosing the tumor was cropped to generate lesion‐centered CT patches. The same preprocessing strategy was applied to both arterial‐phase and portal venous‐phase images. CT intensities were windowed using a soft‐tissue setting and resized to 128 × 128 pixels.

### 4.3 Lesion Foundation Model Construction

The lesion foundation model (LFM) was developed using a DINOv2‐style self‐supervised learning framework [23] to learn transferable lesion‐level CT features (Figure 8A). The backbone network was a ViT‐Small encoder with a patch size of 8, modified to accept single‐channel 128 × 128 CT lesion patches as input. The encoder produced 384D features, which were subsequently used for downstream HCC aggressiveness assessment and recurrence‐free survival prediction.

**FIGURE 8:**
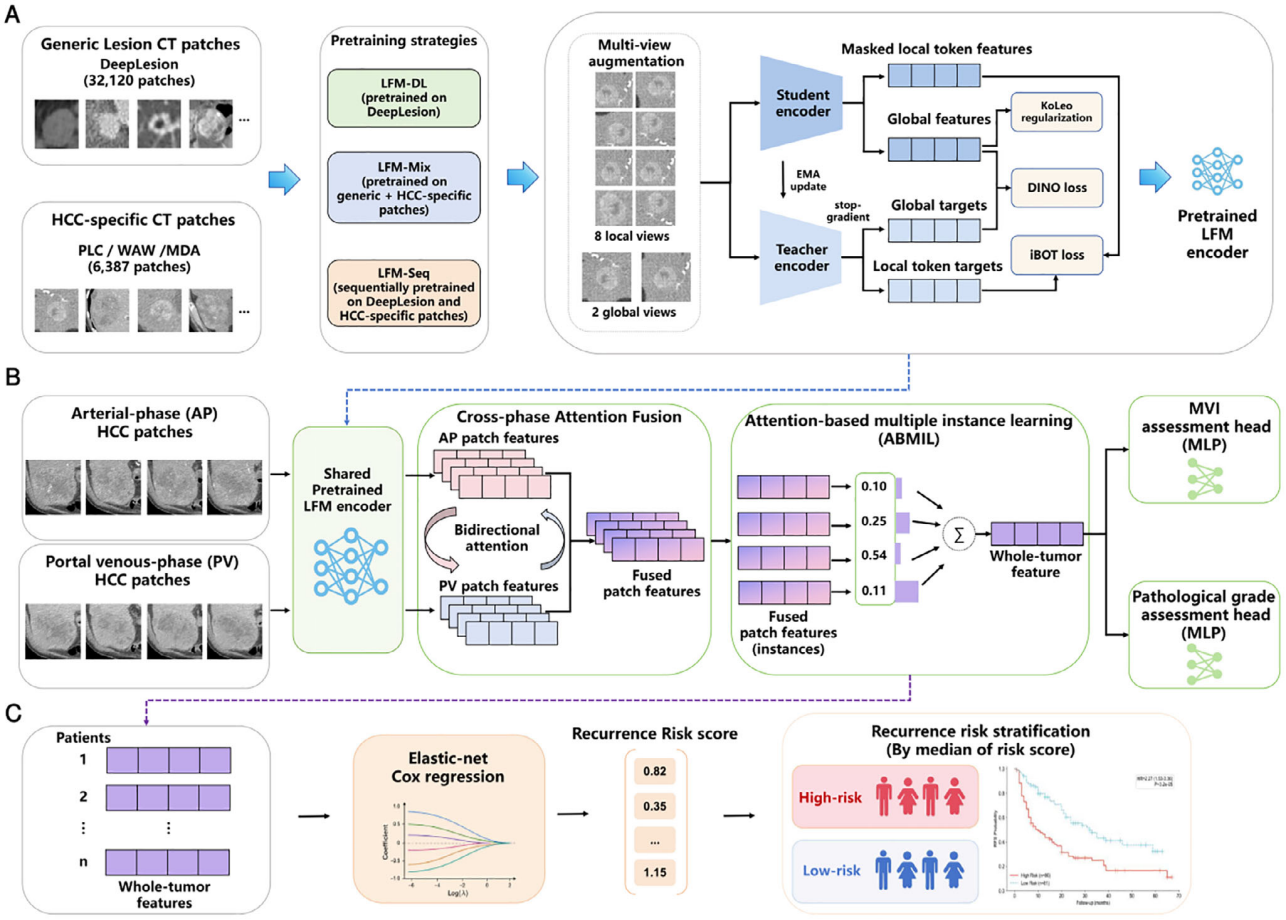
Architecture of the lesion foundation model framework for HCC aggressiveness assessment and RFS prediction. (A) DINOv2 self‐supervised pretraining for lesion foundation model (LFM) construction. (B) LFM‐based HCC aggressiveness assessment. (C) Recurrence‐free survival prediction using LFM‐derived whole‐tumor features.

During CT lesion‐level self‐supervised pretraining, each lesion CT patch was transformed into multiple augmented views, including two global views and eight local views. The global crop size was 128 × 128 pixels, and the local crop size was 96 × 96 pixels. CT‐compatible augmentations, including random horizontal flipping, random vertical flipping and Gaussian blurring, were applied.

Self‐supervised pretraining was performed using a student–teacher self‐distillation framework. The student and teacher networks shared the same ViT architecture. The student network received both global and local augmented views, whereas the teacher network generated target features from global views without gradient backpropagation. The teacher parameters were updated using an exponential moving average of the student parameters, with the momentum coefficient increased according to a cosine schedule from 0.992 to 1.0 during training.

The pretraining objective combined DINO loss, iBOT masked‐token loss and KoLeo regularization. The DINO loss encouraged consistency of global lesion features across different augmented views. The iBOT masked‐token loss promoted learning of local contextual information within lesion CT patches by predicting masked visual tokens. KoLeo regularization was used to improve feature dispersion and reduce the risk of feature collapse. The total loss was defined as the sum of the DINO loss, iBOT loss and KoLeo regularization term.

The CT lesion‐level self‐supervised pretraining was performed using AdamW optimization with an initial learning rate of 0.001, a batch size of 128 and 100 epochs. Weight decay was increased from 0.04 to 0.4 using a cosine schedule. During training, the dispersion of L2‐normalized features was monitored to detect potential feature collapse, and model checkpoints with stable feature dispersion and lower training loss were retained.

To evaluate the impact of different pretraining strategies, four LFM variants were constructed. LFM‐Base used the official DINOv2 pretrained ViT‐Small encoder without additional lesion‐level CT self‐supervised adaptation. LFM‐DL was further pretrained on generic lesion CT patches from DeepLesion. LFM‐Mix was pretrained using a pooled dataset combining generic lesion CT patches and HCC‐specific CT patches. LFM‐Seq was sequentially pretrained by first pretraining the encoder on generic lesion CT patches and then performing a lightweight HCC‐specific adaptation using arterial‐ and portal venous‐phase HCC lesion patches. Because the HCC‐specific patch set was smaller and more homogeneous than the generic lesion pretraining set, the second adaptation stage was limited to one epoch to preserve the generic lesion features and reduce the risk of overfitting or feature instability. The pretrained encoders were subsequently integrated into the downstream tasks for MVI assessment, pathological grade assessment and RFS prediction.

### 4.4 LFM‐Based HCC Aggressiveness Assessment and Robustness Evaluation

The pretrained LFM encoders were integrated into a multitask framework for non‐invasive assessment of HCC aggressiveness, including MVI and pathological grade (Figure 8B). MVI was treated as a binary label according to postoperative pathological diagnosis. Pathological grade was dichotomized as high‐grade HCC (Edmondson–Steiner grade 3/4) and low‐grade HCC (defined as Edmondson–Steiner grade 1/2).

For each patient, arterial‐phase and portal venous‐phase lesion‐centered patches were first processed by a shared pretrained LFM encoder to generate 384D patch‐level features. To integrate complementary enhancement information from arterial and portal venous phases, we introduced a bidirectional cross‐phase attention module to enable feature interaction between phase‐specific patch‐level features. Specifically, arterial‐phase features were updated using contextual information from the corresponding portal venous‐phase features, and vice versa. Residual connections were used to preserve phase‐specific information while incorporating cross‐phase contextual features. The updated arterial‐phase and portal venous‐phase features were then concatenated to form fused patch‐level features.

Attention‐based multiple instance learning (ABMIL) [43] was used to aggregate fused patch‐level features into a patient‐level whole‐tumor feature. A gated attention mechanism was applied to learn the relative contribution of each tumor patch, and the whole‐tumor feature was obtained as the attention‐weighted aggregation of patch‐level features. This feature was then fed into two task‐specific multilayer perceptron heads for MVI assessment and pathological grade assessment.

The downstream aggressiveness assessment model was fine‐tuned in the training cohort from Center 1 and evaluated in the internal validation cohort and two external validation cohorts. All LFM variants were trained using the same fine‐tuning strategy and optimization settings. Model fine‐tuning was performed using binary cross‐entropy loss with uncertainty‐based task weighting to balance the two objectives. AdamW optimization was used with a batch size of 32, weight decay of 1 × 10^−^^4^, and learning rate of 1 × 10^−^^4^. Models were trained for up to 20 epochs using cosine learning‐rate scheduling with a minimum learning rate of 1 × 10^−^^6^ and early stopping with a patience of 5 epochs.

To evaluate the contribution of individual components of LFM‐Seq, ablation experiments were performed under the same fine‐tuning strategy and optimization settings described above. Four ablation variants were constructed: (i) AP_only, using arterial‐phase input only; (ii) PV_only, using portal venous‐phase input only; (iii) simple_fusion, replacing the bidirectional cross‐phase attention module with direct concatenation of phase‐specific features; and (iv) mean_pool and max_pool, replacing the ABMIL aggregation with mean or max pooling of patch‐level features. AUCs were computed on the best checkpoint of each variant, and each variant was compared with the full LFM‐Seq model on the same cohort using the DeLong test.

To evaluate the robustness of HCC aggressiveness assessment under clinically relevant conditions, two additional analyses were performed. First, a tumor‐localization perturbation analysis was conducted to simulate variation in lesion localization. Tumor bounding boxes were randomly perturbed at predefined levels from 0% to 25% with 5% increment, and model performance was repeatedly evaluated 50 times under each perturbation level. This analysis was designed to assess whether the assessment remained stable when tumor localization was imprecise. Second, a limited‐training‐data analysis was performed by retraining models using reduced proportions of the training cohort, including 10%, 20% and 50% of the available training data and each setting was repeated 50 times.

All deep learning experiments were implemented in Python using PyTorch 2.5.1 and performed on an NVIDIA H800 GPU.

### 4.5 Recurrence‐Free Survival Prediction Using LFM‐Derived Whole‐Tumor Features

To evaluate whether LFM‐derived whole‐tumor features could provide prognostic information beyond pathological aggressiveness assessment, we further used these features for RFS prediction (Figure 8C). For each patient, arterial‐phase and portal venous‐phase tumor patches were processed through the fine‐tuned LFM model, including the shared LFM encoder, cross‐phase attention module and ABMIL aggregation module, resulting in a patient‐level whole‐tumor feature.

RFS modeling was performed in the two external cohorts with available postoperative follow‐up. EVC1 was used as the RFS model development cohort, and EVC2 was used as the independent validation cohort. LFM‐derived whole‐tumor features were used as candidate prognostic imaging features. To reduce dimensionality, univariable Cox regression was first performed for feature pre‐screening, after which the retained features were entered into an elastic‐net Cox proportional hazards model. The elastic‐net penalty was used to handle high‐dimensional imaging features and reduce overfitting by combining feature selection with coefficient shrinkage. The penalty parameter and mixing parameter were selected by five‐fold cross‐validation according to the concordance index. Feature pre‐screening was performed within each fold using only the training partitions. The final model was refitted on the entire EVC1 cohort with the selected hyperparameters. EVC2 was not involved in feature selection, hyperparameter tuning, or cutoff determination.

For recurrence risk stratification, patients were categorized into high‐risk and low‐risk groups using the median risk score in EVC1 as the cutoff. This cutoff was fixed and directly applied to EVC2 to avoid cohort‐specific threshold optimization and to enable independent validation of risk stratification performance.

For comparison, two clinical reference models were constructed using the same elastic‐net Cox framework without univariable feature pre‐screening, given the limited number of clinical features. The Clinical model was built using eight variables available before surgery (age, sex, tumor size, HBsAg, AFP, ALT, AST, and GGT), reflecting the information accessible at the point of preoperative decision‐making, at which MVI and pathological grade are unknown. The Clinicopathological model additionally incorporated the postoperative pathological variables (MVI and pathological grade).

### 4.6 Visual and Biological Interpretation Analysis

To assess whether LFM‐derived outputs were spatially and biologically plausible, we performed visual interpretation for HCC aggressiveness assessment and imaging‐genomic analysis for LFM‐derived recurrence risk scores.

For visual interpretation, occlusion‐based heatmaps [44] were generated to identify image regions contributing to LFM‐based MVI and pathological grade assessment. For each lesion patch, an 8 × 8 occlusion window was systematically moved across the patch with a stride of 8 pixels, and the change in model output after occlusion was recorded. Heatmaps were generated with respect to the positive class for each task, namely MVI‐positive probability for MVI assessment and high‐grade probability for pathological grade assessment. Regions where occlusion caused a larger decrease in the predicted probability were considered to have a greater contribution to positive class. For quantitative region‐level attribution analysis, regions with occlusion contributions ≥ 50% of the maximum contribution within each attribution map were defined as high‐contribution regions. For each patient, the proportion of high‐contribution pixels overlapping the tumor mask was calculated. To evaluate contributions adjacent to the tumor boundary, the tumor mask was additionally expanded by 10 pixels and the corresponding overlap was calculated. These analyses were performed separately for AP and PV images. Within‐patient differences in spatial overlap between AP and PV were assessed using the Wilcoxon signed‐rank test.

For biological interpretation, the matched TCGA‐LIHC/TCIA imaging‐genomic cohort was used to explore the biological relevance of LFM‐derived recurrence risk scores. Multiphase CT images from TCGA‐LIHC were processed using the same preprocessing pipeline and passed through the fine‐tuned LFM model to extract whole‐tumor features. The elastic‐net Cox model developed in EVC1 was then applied to these features to calculate a risk score for each patient. Patients in the biological analysis cohort were stratified into LFM‐derived high‐risk and low‐risk groups according to the median risk score cutoff from EVC1.

To investigate the biological basis of the LFM‐derived recurrence risk phenotype, we performed an imaging‐genomic association analysis using the TCGA‐LIHC cohort with matched contrast‐enhanced CT imaging and RNA‐sequencing data. Patients were stratified into high‐risk and low‐risk groups using the fixed cutoff derived from EVC1. Genes with consistently low read counts across samples were filtered before differential expression analysis. Differentially expressed genes (DEGs) between the two risk groups were identified using DESeq2 with the Wald test based on raw RNA‐sequencing count data [45], and log2 fold changes were shrunk using the apeglm method [46]. DEGs were defined using a false discovery rate (FDR) < 0.10 and an absolute log2 fold change > 0.5.

Gene Ontology biological process enrichment analysis was performed separately for genes upregulated and downregulated in the high‐risk group using clusterProfiler [47], with hypergeometric testing and Benjamini–Hochberg FDR correction. All expressed genes retained after filtering were used as the background gene set. To characterize risk‐associated molecular interaction modules, protein–protein interaction networks were constructed from differentially expressed genes using the STRING database (version 12.0) [48] with interactions retained at a combined score ≥ 700. Hub genes were identified by maximal clique centrality ranking using cytoHubba [49].

Immune‐cell composition was estimated from bulk RNA‐sequencing TPM data using CIBERSORT with the LM22 leukocyte signature matrix [50, 51]. CIBERSORT was run using ν‐support vector regression with 100 permutations, and quantile normalization was disabled for RNA‐sequencing data. Differences in estimated immune‐cell fractions between risk groups were assessed using the two‐sided Wilcoxon rank‐sum test with Benjamini–Hochberg FDR correction. Given the limited sample size of the matched imaging‐genomic cohort, immune‐cell differences were interpreted as exploratory associations.

### 4.7 Statistical Analysis

Continuous variables were summarized as mean ± standard deviation or median with interquartile range, as appropriate, and categorical variables were summarized as counts and percentages. Baseline characteristics were compared among cohorts using one‐way analysis of variance or the Kruskal‐Wallis test for continuous variables and the chi‐square test or Fisher's exact test for categorical variables, as appropriate.

For MVI and pathological grade assessment, model discrimination was evaluated using the area under the receiver operating characteristic curve (AUC). The classification threshold was determined on the training cohort by maximizing the Youden index and was then applied unchanged to the internal and external validation cohorts. Sensitivity, specificity, positive predictive value and negative predictive value were also calculated. The performance of LFM variants, including LFM‐Base, LFM‐DL, LFM‐Mix and LFM‐Seq, was compared with the radiomics model and the 3D lesion foundation model FMCIB [21]. Implementation details of the Radiomics model and FMCIB are provided in the Supporting Information.

Differences in AUCs between models were assessed using the DeLong test. Calibration curves were used to evaluate agreement between predicted probabilities and observed outcomes, and the Brier scores, calibration intercepts, and calibration slopes were also reported. The decision curve analysis was performed to assess clinical net benefit across threshold probabilities.

For RFS prediction, model discrimination was evaluated using the concordance index (C‐index) and time‐dependent AUCs. Differences in C‐index and time‐dependent AUCs between models were assessed using bootstrap‐based comparisons. Kaplan–Meier curves were used to compare RFS between high‐risk and low‐risk groups, and differences were assessed using the log‐rank test.

Univariable and multivariable Cox proportional hazards regression analyses were performed separately in EVC1 and EVC2 to evaluate the association between LFM‐derived risk scores and RFS. The proportional‐hazards assumption was assessed using scaled Schoenfeld residuals (per‐variable and global tests). For the LFM‐Seq risk score, time‐dependent hazard ratios were additionally estimated using Cox models with a log‐time interaction term, and these are reported when the assumption was violated. Median follow‐up was estimated using the reverse Kaplan–Meier method. The risk score was standardized to a mean of 0 and standard deviation (SD) of 1 using the mean and SD of EVC1, and hazard ratios are reported per 1 SD increment, using EVC1 as the reference for standardization. Multivariable models were adjusted for clinicopathological features.

Incremental prognostic value was further assessed using integrated discrimination improvement (IDI) and net reclassification improvement (NRI) analyses. Two nested comparisons were performed: (1) the preoperative clinical model versus the clinical model plus the LFM‐Seq risk score, to evaluate incremental value beyond routinely available preoperative clinical factors; and (2) the clinicopathological model versus the clinicopathological model plus the LFM‐Seq risk score, to evaluate incremental value beyond conventional clinicopathological factors, including MVI and pathological grade.

The code associated with this work is publicly available on GitHub at the repository https://github.com/xiawei999000/LFM‐HCC.

## Author Contributions

**Wei Xia**: methodology, conceptualization, data curation, investigation, validation, writing – original draft, writing – review and editing. **Zhehan Shen**: methodology, conceptualization, data curation, formal analysis, writing – original draft, writing – review and editing, visualization, investigation. **Mengsi Li**: data curation, conceptualization, writing – original draft, writing – review and editing. **Jianxin Lu**: writing – review and editing, writing – original draft, data curation. **Ji Xia**: data curation, writing – original draft, writing – review and editing. **Yang Cheng**: data curation, writing – original draft, writing – review and editing. **Wenzheng Li**: data curation, resources, writing – original draft, writing – review and editing. **Shunjie Dong**: conceptualization, methodology, project administration, writing – original draft, writing – review and editing, resources. **Lin Deng**: conceptualization, data curation, formal analysis, project administration, writing – original draft, writing – review and editing, resources. **Ruokun Li**: conceptualization, methodology, data curation, investigation, supervision, funding acquisition, project administration, resources, writing – original draft, writing – review and editing.

## Conflicts of Interest

The authors declare no conflicts of interest.

## Supporting information




**Supporting File**: advs78587‐sup‐0001‐SuppMat.docx.

## Data Availability

The data that support the findings of this study are openly available in Pretraining dataset and model weights at https://zenodo.org/records/20710784.

## References

[1] F. Bray , M. Laversanne , H. Sung , et al., “Global Cancer Statistics 2022: GLOBOCAN Estimates of Incidence and Mortality Worldwide for 36 Cancers in 185 Countries,” CA: A Cancer Journal for Clinicians 74 (2024): 229–263, 10.3322/caac.21834. 38572751

[2] A. G. Singal , J. M. Llovet , M. Yarchoan , et al., “AASLD Practice Guidance on Prevention, Diagnosis, and Treatment of Hepatocellular Carcinoma,” Hepatology 78, no. 6 (2023): 1922–1965, 10.1097/HEP.0000000000000466. PMC10663390 37199193

[3] B. Sangro , J. Argemi , M. Ronot , et al., “EASL Clinical Practice Guidelines on the Management of Hepatocellular Carcinoma,” Journal of Hepatology 82, no. 2 (2025): 315–374, 10.1016/j.jhep.2024.08.028. 39690085

[4] J. M. Llovet , R. K. Kelley , A. Villanueva , et al., “Hepatocellular Carcinoma,” Nature Reviews Disease Primers 7, no. 1 (2021): 6, 10.1038/s41572-020-00240-3. PMC13537433 33479224

[5] X.‐F. Xu , H. Xing , J. Han , et al., “Risk Factors, Patterns, and Outcomes of Late Recurrence after Liver Resection for Hepatocellular Carcinoma,” JAMA Surgery 154, no. 3 (2019): 209, 10.1001/jamasurg.2018.4334. PMC6439634 30422241

[6] H. Jiang , B. Li , T. Zheng , et al., “MRI‐Based Prediction of Microvascular Invasion/High Tumor Grade and Adjuvant Therapy Benefit for Solitary HCC ≤ 5 cm: A Multicenter Cohort Study,” European Radiology 35, no. 6 (2024): 3223–3237, 10.1007/s00330-024-11295-1. PMC12081510 39702639

[7] D. Shi , Q. Kou , H.‐Y. Chen , et al., “Quantitative Analysis of Enhanced CT in Predicting Microvascular Invasion and Pathological Grading of Hepatocellular Carcinoma,” European Radiology 36, no. 4 (2025): 2778–2793, 10.1007/s00330-025-12087-x. 41171351

[8] R. Stigliano , L. Marelli , D. Yu , N. Davies , D. Patch , and A. K. Burroughs , “Seeding Following Percutaneous Diagnostic and Therapeutic Approaches for Hepatocellular Carcinoma. What Is the Risk and the Outcome?,” Cancer Treatment Reviews 33, no. 5 (2007): 437–447, 10.1016/j.ctrv.2007.04.001. 17512669

[9] V. Chernyak , K. J. Fowler , A. Kamaya , et al., “Liver Imaging Reporting and Data System (LI‐RADS) Version 2018: Imaging of Hepatocellular Carcinoma in at‐Risk Patients,” Radiology 289, no. 3 (2018): 816–830, 10.1148/radiol.2018181494. PMC6677371 30251931

[10] M. Ronot , V. Chernyak , A. Burgoyne , et al., “Imaging to Predict Prognosis in Hepatocellular Carcinoma: Current and Future Perspectives,” Radiology 307, no. 3 (2023): 221429, 10.1148/radiol.221429. 37014244

[11] J. H. Min , M. W. Lee , H. S. Park , et al., “Interobserver Variability and Diagnostic Performance of Gadoxetic Acid–Enhanced MRI for Predicting Microvascular Invasion in Hepatocellular Carcinoma,” Radiology 297, no. 3 (2020): 573–581, 10.1148/radiol.2020201940. 32990512

[12] Y. Jiang , K. Wang , Y.‐R. Wang , et al., “Preoperative and Prognostic Prediction of Microvascular Invasion in Hepatocellular Carcinoma: A Review Based on Artificial Intelligence,” Technology in Cancer Research & Treatment 22 (2023): 15330338231212726, 10.1177/15330338231212726. PMC10631353 37933176

[13] T.‐Y. Xia , Z.‐H. Zhou , X.‐P. Meng , et al., “Predicting Microvascular Invasion in Hepatocellular Carcinoma Using CT‐Based Radiomics Model,” Radiology 307, no. 4 (2023): 222729, 10.1148/radiol.222729. 37097141

[14] X. Xu , H.‐L. Zhang , Q.‐P. Liu , et al., “Radiomic Analysis of Contrast‐Enhanced CT Predicts Microvascular Invasion and Outcome in Hepatocellular Carcinoma,” Journal of Hepatology 70, no. 6 (2019): 1133–1144, 10.1016/j.jhep.2019.02.023. 30876945

[15] G.‐W. Ji , F.‐P. Zhu , Q. Xu , et al., “Machine‐Learning Analysis of Contrast‐Enhanced CT Radiomics Predicts Recurrence of Hepatocellular Carcinoma after Resection: A Multi‐Institutional Study,” EBioMedicine 50 (2019): 156–165, 10.1016/j.ebiom.2019.10.057. PMC6923482 31735556

[16] X. Chen , Y. Tang , D. Wu , et al., “From Imaging to Clinical Outcome: Dual‐Region CT Radiomics Predicting FOXM1 Expression and Prognosis in Hepatocellular Carcinoma,” Frontiers in Oncology 13 (2023): 1278467, 10.3389/fonc.2023.1278467. PMC10561750 37817774

[17] P. Lambin , R. T. H. Leijenaar , T. M. Deist , et al., “Radiomics: The Bridge between Medical Imaging and Personalized Medicine,” Nature Reviews Clinical Oncology 14, no. 12 (2017): 749–762, 10.1038/nrclinonc.2017.141. 28975929

[18] W. Zhang , Q. Guo , Y. Zhu , et al., “Cross‐Institutional Evaluation of Deep Learning and Radiomics Models in Predicting Microvascular Invasion in Hepatocellular Carcinoma: Validity, Robustness, and Ultrasound Modality Efficacy Comparison,” Cancer Imaging 24, no. 1 (2024): 142, 10.1186/s40644-024-00790-9. PMC11520182 39438929

[19] Z. Huang , W. Huang , L. Jiang , et al., “Decision Fusion Model for Predicting Microvascular Invasion in Hepatocellular Carcinoma Based on Multi‐MR Habitat Imaging and Machine‐Learning Classifiers,” Academic Radiology 32, no. 4 (2025): 1971–1980, 10.1016/j.acra.2024.10.007. 39472207

[20] M. Moor , O. Banerjee , Z. S. H. Abad , et al., “Foundation Models for Generalist Medical Artificial Intelligence,” Nature 616, no. 7956 (2023): 259–265, 10.1038/s41586-023-05881-4. 37045921

[21] S. Pai , D. Bontempi , I. Hadzic , et al., “Foundation Model for Cancer Imaging Biomarkers,” Nature Machine Intelligence 6, no. 3 (2024): 354–367, 10.1038/s42256-024-00807-9. PMC10957482 38523679

[22] J. Ma , Y. He , F. Li , L. Han , C. You , and B. Wang , “Segment Anything in Medical Images,” Nature Communications 15, no. 1 (2024): 654, 10.1038/s41467-024-44824-z. PMC10803759 38253604

[23] M. Oquab , T. Darcet , and T. Moutakanni , et al., “DINOv2: Learning Robust Visual Features without Supervision” Transactions on Machine Learning Research (2024), https://openreview.net/forum?id=a68SUt6zFt.

[24] H. Xu , N. Usuyama , J. Bagga , et al., “A Whole‐Slide Foundation Model for Digital Pathology from Real‐World Data,” Nature 630, no. 8015 (2024): 181–188, 10.1038/s41586-024-07441-w. PMC11153137 38778098

[25] E. Vorontsov , A. Bozkurt , A. Casson , et al., “A Foundation Model for Clinical‐Grade Computational Pathology and Rare Cancers Detection,” Nature Medicine 30, no. 10 (2024): 2924–2935, 10.1038/s41591-024-03141-0. PMC11485232 39039250

[26] T. Ding , S. J. Wagner , A. H. Song , et al., “A Multimodal Whole‐Slide Foundation Model for Pathology,” Nature Medicine 31, no. 11 (2025): 3749–3761, 10.1038/s41591-025-03982-3. PMC12618242 41193692

[27] T. Xu , S. Hosseini , C. Anderson , et al., “A Generalizable 3D Framework and Model for Self‐Supervised Learning in Medical Imaging,” npj Digital Medicine 8, no. 1 (2025): 639, 10.1038/s41746-025-02035-w. PMC12594808 41203891

[28] K. Yan , X. Wang , L. Lu , and R. M. Summers , “DeepLesion: Automated Mining of Large‐Scale Lesion Annotations and Universal Lesion Detection with Deep Learning,” Journal of Medical Imaging 5, no. 03 (2018): 1–11, 10.1117/1.JMI.5.3.036501. PMC6052252 30035154

[29] Y.‐B. Zhang , Z.‐Q. Chen , Y. Bu , P. Lei , W. Yang , and W. Zhang , “Construction of a 2.5D Deep Learning Model for Predicting Early Postoperative Recurrence of Hepatocellular Carcinoma Using Multi‐View and Multi‐Phase CT Images,” Journal of Hepatocellular Carcinoma 11 (2024): 2223–2239, 10.2147/JHC.S493478. PMC11577935 39569409

[30] J. Zhu , B. Xu , T. Fan , et al., “Sub‐Regional Radiomics Combining Multichannel 2‐Dimensional or 3‐Dimensional Deep Learning for Predicting Neoadjuvant Chemo‐Immunotherapy Response in Esophageal Squamous Cell Carcinoma: A Multicenter Study,” npj Precision Oncology 9, no. 1 (2025): 248, 10.1038/s41698-025-01047-9. PMC12279934 40691312

[31] A. Sharma , J. J. W. Seow , C.‐A. Dutertre , et al., “Onco‐Fetal Reprogramming of Endothelial Cells Drives Immunosuppressive Macrophages in Hepatocellular Carcinoma,” Cell 183, no. 2 (2020): 377–394.e21, 10.1016/j.cell.2020.08.040. 32976798

[32] D. Li , T. Zhang , Y. Guo , C. Bi , M. Liu , and G. Wang , “Biological Impact and Therapeutic Implication of Tumor‐Associated Macrophages in Hepatocellular Carcinoma,” Cell Death & Disease 15, no. 7 (2024): 498, 10.1038/s41419-024-06888-z. PMC11245522 38997297

[33] Q. Feng , H. Lu , and L. Wu , “Identification of M2‐Like Macrophage‐Related Signature for Predicting the Prognosis, Ecosystem and Immunotherapy Response in Hepatocellular Carcinoma,” PLoS ONE 18, no. 9 (2023): 0291645, 10.1371/journal.pone.0291645. PMC10508629 37725627

[34] Z.‐C. Jin , J. Wei , Y.‐D. Xiao , et al., “Decoding Tumor Heterogeneity with Imaging Biomarkers Predicts Response to TACE plus Immunotherapy and Targeted Therapy in HCC (CHANCE2204),” Hepatology 84 (2025): 1316, 10.1097/HEP.0000000000001593. PMC13668028 41213031

[35] Y. Xie , F. Wang , J. Wei , et al., “Noninvasive Prognostic Classification of ITH in HCC with Multi‐Omics Insights and Therapeutic Implications,” Science Advances 11 (2025): ads8323, 10.1126/sciadv.ads8323. PMC12047409 40315307

[36] S. Lee , T. W. Kang , K. D. Song , et al., “Effect of Microvascular Invasion Risk on Early Recurrence of Hepatocellular Carcinoma after Surgery and Radiofrequency Ablation,” Annals of Surgery 273, no. 3 (2021): 564–571, 10.1097/SLA.0000000000003268. 31058694

[37] X.‐P. Meng , T.‐Y. Tang , Z.‐M. Ding , et al., “Preoperative Microvascular Invasion Prediction to Assist in Surgical Plan for Single Hepatocellular Carcinoma: Better Together with Radiomics,” Annals of Surgical Oncology 29, no. 5 (2022): 2960–2970, 10.1245/s10434-022-11346-1. 35102453

[38] J. Luo , X. Wan , J. Du , et al., “Comprehensive Multi‐Phase 3D Contrast‐Enhanced CT Imaging for Primary Liver Cancer,” Scientific Data 12, no. 1 (2025): 768, 10.1038/s41597-025-05125-2. PMC12065907 40348741

[39] K. Bartnik , T. Bartczak , M. Krzyziński , et al., “WAW‐TACE: A Hepatocellular Carcinoma Multiphase CT Dataset with Segmentations, Radiomics Features, and Clinical Data,” Radiology: Artificial Intelligence 6 (2024): 240296, 10.1148/ryai.240296. PMC11605144 39441110

[40] A. W. Moawad , A. Morshid , A. M. Khalaf , et al., “Multimodality Annotated Hepatocellular Carcinoma Data Set Including Pre‐ and Post‐TACE with Imaging Segmentation,” Scientific Data 10, no. 1 (2023): 33, 10.1038/s41597-023-01928-3. PMC9849450 36653372

[41] A. Ally , M. Balasundaram , R. Carlsen , et al., “Comprehensive and Integrative Genomic Characterization of Hepatocellular Carcinoma,” Cell 169, no. 7 (2017): 1327–1341.e23, 10.1016/j.cell.2017.05.046. PMC5680778 28622513

[42] K. Clark , B. Vendt , K. Smith , et al., “The Cancer Imaging Archive (TCIA): Maintaining and Operating a Public Information Repository,” Journal of Digital Imaging 26, no. 6 (2013): 1045–1057, 10.1007/s10278-013-9622-7. PMC3824915 23884657

[43] M. Ilse , J. M. Tomczak , and M. Welling , “Attention‐based Deep Multiple Instance Learning,” in Proceedings of the 35th International Conference on Machine Learning (2018): 2127–2136, https://proceedings.mlr.press/v80/ilse18a.html.

[44] D. S. Kermany , M. Goldbaum , W. Cai , et al., “Identifying Medical Diagnoses and Treatable Diseases by Image‐Based Deep Learning,” Cell 172, no. 5 (2018): 1122–1131.e9, 10.1016/j.cell.2018.02.010. 29474911

[45] M. I. Love , W. Huber , and S. Anders , “Moderated Estimation of Fold Change and Dispersion for RNA‐seq Data with DESeq2,” Genome Biology 15, no. 12 (2014): 550, 10.1186/s13059-014-0550-8. PMC4302049 25516281

[46] A. Zhu , J. G. Ibrahim , and M. I. Love , “Heavy‐Tailed Prior Distributions for Sequence Count Data: Removing the Noise and Preserving Large Differences,” Bioinformatics 35, no. 12 (2019): 2084–2092, 10.1093/bioinformatics/bty895. PMC6581436 30395178

[47] T. Wu , E. Hu , S. Xu , et al., “clusterProfiler 4.0: A Universal Enrichment Tool for Interpreting Omics Data,” The Innovation 2, no. 3 (2021): 100141, 10.1016/j.xinn.2021.100141. PMC8454663 34557778

[48] D. Szklarczyk , R. Kirsch , M. Koutrouli , et al., “The STRING Database in 2023: Protein–protein Association Networks and Functional Enrichment Analyses for any Sequenced Genome of Interest,” Nucleic Acids Research 51, no. D1 (2023): D638–D646, 10.1093/nar/gkac1000. PMC9825434 36370105

[49] C.‐H. Chin , S.‐H. Chen , H.‐H. Wu , C.‐W. Ho , M.‐T. Ko , and C.‐Y. Lin , “cytoHubba: Identifying Hub Objects and Sub‐Networks from Complex Interactome,” BMC Systems Biology 8, no. S4 (2014): S11, 10.1186/1752-0509-8-S4-S11. PMC4290687 25521941

[50] A. M. Newman , C. L. Liu , M. R. Green , et al., “Robust Enumeration of Cell Subsets from Tissue Expression Profiles,” Nature Methods 12, no. 5 (2015): 453–457, 10.1038/nmeth.3337. PMC4739640 25822800

[51] A. M. Newman , C. B. Steen , C. L. Liu , et al., “Determining Cell Type Abundance and Expression from Bulk Tissues with Digital Cytometry,” Nature Biotechnology 37, no. 7 (2019): 773–782, 10.1038/s41587-019-0114-2. PMC6610714 31061481

