# Supplementary material for: A CT Lesion Foundation Model for Non‐Invasive Assessment of Hepatocellular Carcinoma Aggressiveness and Prediction of Recurrence Risk
Source: Adv Sci (Weinh). 2026 Nov 1:e78587. Online ahead of print. doi: 10.1002/advs.78587 (PMC13723727; doi:10.1002/advs.78587)
Supplement: Supplementary file 1 [file ADVS-9999-e78587-s001.docx]

A. **Supplementary methods**

**CT Imaging protocol**

The CT imaging protocols of the three centers were as follows. All patients underwent unenhanced and contrast-enhanced multiphasic abdominal CT scans, using one of the following systems: PHILIPS iCT 256, PHILIPS IQon, GE Revolution CT, GE Discovery CT750 HD, GE LightSpeed 16, GE LightSpeed VCT, TOSHIBA Aquilion ONE, SIEMENS SOMATOM Definition, SIEMENS SOMATOM Definition Flash, SIEMENS SOMATOM Force, UIH uCT 960+, and UIH uCT 760. Scanning parameters were as follows: tube voltage of 100 or 120 kVp; tube current determined by automatic exposure control with a standard deviation setting; matrix, 512 × 512; slice thickness of 5 mm. A 1.5-2.0 mL/kg body weight bolus of iodinated contrast media was injected intravenously at a flow rate of 3.0–4.0 mL/sec, followed by a 20-mL saline chaser bolus injection. Arterial phase scanning was initiated with a delay of approximately 20 seconds after enhancement of the descending aorta to 100 HU, as measured using a bolus-tracking technique; portal venous and delayed phase images were obtained at 70 and 180 seconds, respectively, after contrast injection.

**Radiomics model of HCC aggressiveness assessment**

Radiomics features were extracted from arterial-phase (AP) and portal venous-phase (PV) CT images using the manually delineated three-dimensional tumor masks. Feature extraction was performed with the PyRadiomics package (version 3.0.1) following the Image Biomarker Standardisation Initiative recommendations. Prior to feature extraction, all images were resampled to isotropic voxel spacing and intensity values were discretized using a fixed bin width to reduce scanner-related variability. For each phase, radiomics features were extracted from both the original images and wavelet-transformed images generated using eight three-dimensional wavelet decompositions (LLL, LLH, LHL, LHH, HLL, HLH, HHL, and HHH). The extracted features included first-order intensity statistics, shape descriptors, gray-level co-occurrence matrix (GLCM), gray-level run-length matrix (GLRLM), gray-level size-zone matrix (GLSZM), gray-level dependence matrix (GLDM), and neighboring gray-tone difference matrix (NGTDM) features. In total, more than one thousand radiomics features were generated for each imaging phase. Features extracted from AP and PV images were concatenated to form a comprehensive multiphase radiomics representation for each patient.

Radiomics models were developed independently for MVI and pathological grade assessment. To avoid information leakage, all feature preprocessing, selection, and model fitting procedures were performed exclusively within the training cohort. First, features with zero or near-zero variance were removed. Subsequently, pairwise Pearson correlation analysis was conducted, and among highly correlated feature pairs (absolute correlation coefficient > 0.90), redundant features were discarded. The remaining features were ranked using the minimum redundancy maximum relevance (mRMR) algorithm, which simultaneously maximizes feature relevance to the target outcome while minimizing inter-feature redundancy. The top 30 ranked features were retained as candidate predictors. Final feature selection and classifier construction were performed using least absolute shrinkage and selection operator (LASSO) regression. The regularization parameter was optimized through five-fold stratified cross-validation within the training cohort, and the value corresponding to the minimum cross-validation error was selected. Features with non-zero coefficients after LASSO regularization were retained to construct the final radiomics model.

**FMCIB foundation model fine-tuning for HCC aggressiveness assessment**

Lesion-centered three-dimensional AP and PV CT patches were used as inputs to a 3D lesion foundation model based on the publicly available foundation model for cancer imaging biomarker discovery (FMCIB). All 3D patches were resampled to 50×50×50 voxels and normalized by clipping CT attenuation values to 0-400 HU and scaling them to [0,1]. FMCIB backbones were used for AP and PV, and the extracted phase-specific deep features were projected to a 512-dimensional feature space. The same bidirectional cross-phase attention module from LFM fine-tuning framework was then used to fuse complementary enhancement information between the two phases. The fused features were concatenated and passed to two task-specific multilayer perceptron heads for MVI and pathological grade assessment.

The FMCIB baseline was fine-tuned using a multitask binary cross-entropy loss with uncertainty-based task weighting. Training was performed with the AdamW optimizer (initial learning rate = 1×10^-4^, weight decay = 1×10^-5^), a batch size of 32, and cosine annealing learning-rate scheduling. The pretrained FMCIB backbone and classification heads were optimized end-to-end for a maximum of 100 epochs, with early stopping applied if the validation loss did not improve for 15 consecutive epochs. Dropout with a rate of 0.3 was applied in the task-specific multilayer perceptron heads to reduce overfitting.

**Radiomics and FMCIB-based RFS prediction model**

Radiomics- and FMCIB-based RFS prediction models were constructed as baseline survival models using the same development and validation strategy as the LFM-derived RFS model. For the radiomics-based RFS prediction, handcrafted multiphase radiomics features extracted from arterial-phase and portal venous-phase tumor regions were used as input features. For the FMCIB-based RFS prediction, deep imaging features derived from lesion-centered three-dimensional arterial-phase and portal venous-phase CT patches using the FMCIB-based model were used as input features. For each baseline feature set, model development was performed in EVC1 and independent validation was performed in EVC2.

B. **Supplementary figures**

**Figure S1. The data inclusion and exclusion flow diagram.** The study included three parts. First, generic lesion CT patches from DeepLesion and HCC-specific CT patches from PLC, WAW and MDA cohorts were used for self-supervised pretraining of the LFM. Second, patients with HCC who underwent hepatectomy were retrospectively collected from three centers for aggressiveness assessment. After exclusion, Center 1 was divided into a training cohort and an internal validation cohort, while Centers 2 and 3 served as two external validation cohorts. Third, external validation cohorts 1 and 2 were used for RFS prediction model development and validation, respectively. The TCGA-LIHC cohort was screened to obtain the biological analysis set.

**Figure S2. The AUC of models in MVI assessment across tumor size subgroups.**

**Figure S3. The AUC of models in pathological grade assessment across tumor size subgroups.**

**Figure S4. The C-index of models in RFS prediction across tumor size subgroups.**

**Figure S5. The time-dependent AUC of models in RFS prediction across tumor size subgroups.**

**Figure S6. Region-level occlusion analysis of the LFM-Seq model for MVI and pathological grade assessment. (A) Two representative MVI-positive patients and (B) two representative high-grade patients, shown on the axial slice with the largest tumor area. For each patient, the arterial (AP) and portal venous (PV) CT images are displayed side by side with the corresponding occlusion heatmaps. Red contours delineate the tumor mask; dashed yellow contours delineate the high-contribution regions (occlusion blocks with contribution ≥ 50% of the maximum).**

C. **Supplementary tables**

**Table S1. Performance of MVI assessment.**

| **Cohort** | **Model** | **AUC**  **(95% CI)** | **Sensitivity (95% CI)** | **Specificity (95% CI)** | **PPV**  **(95% CI)** | **NPV**  **(95% CI)** |
| --- | --- | --- | --- | --- | --- | --- |
| IVC | LFM-Base | 0.661  (0.572–0.746) | 0.841  (0.614–0.956) | 0.536  (0.398–0.745) | 0.451  (0.359–0.575) | 0.881  (0.786–0.965) |
|  | LFM-DL | 0.894  (0.837–0.942) | 0.773  (0.684–0.972) | 0.887  (0.639–0.948) | 0.756  (0.528–0.881) | 0.896  (0.849–0.984) |
|  | LFM-Mix | 0.852  (0.778–0.918) | 0.886  (0.674–0.974) | 0.722  (0.667–0.931) | 0.591  (0.507–0.842) | 0.933  (0.846–0.986) |
|  | LFM-Seq | 0.916  (0.870–0.957) | 0.886  (0.826–1.000) | 0.845  (0.682–0.922) | 0.722  (0.564–0.855) | 0.943  (0.911–1.000) |
|  | FMCIB | 0.749  (0.657–0.841) | 0.545  (0.415–0.911) | 0.876  (0.500–0.943) | 0.667  (0.402–0.824) | 0.810  (0.743–0.932) |
|  | Radiomics | 0.802  (0.725–0.869) | 0.750  (0.658–1.000) | 0.732  (0.466–0.862) | 0.559  (0.402–0.717) | 0.866  (0.821–1.000) |
| EVC1 | LFM-Base | 0.596  (0.514–0.676) | 0.573  (0.414–0.903) | 0.646  (0.284–0.804) | 0.558  (0.460–0.682) | 0.660  (0.584–0.813) |
|  | LFM-DL | 0.825  (0.759–0.884) | 0.920  (0.803–0.976) | 0.677  (0.587–0.816) | 0.690  (0.616–0.805) | 0.915  (0.818–0.973) |
|  | LFM-Mix | 0.797  (0.728–0.863) | 0.747  (0.667–0.949) | 0.760  (0.539–0.865) | 0.709  (0.580–0.821) | 0.793  (0.738–0.940) |
|  | LFM-Seq | 0.846  (0.784–0.900) | 0.960  (0.916–1.000) | 0.698  (0.589–0.802) | 0.713  (0.619–0.804) | 0.957  (0.913–1.000) |
|  | FMCIB | 0.786  (0.713–0.852) | 0.707  (0.535–0.845) | 0.781  (0.657–0.926) | 0.716  (0.623–0.873) | 0.773  (0.684–0.871) |
|  | Radiomics | 0.811  (0.749–0.873) | 0.827  (0.718–0.986) | 0.698  (0.495–0.822) | 0.681  (0.568–0.792) | 0.838  (0.768–0.982) |
| EVC2 | LFM-Base | 0.640  (0.560–0.729) | 0.681  (0.344–0.931) | 0.562  (0.307–0.904) | 0.667  (0.583–0.833) | 0.577  (0.481–0.807) |
|  | LFM-DL | 0.905  (0.859–0.948) | 0.777  (0.725–0.967) | 0.890  (0.689–0.955) | 0.901  (0.786–0.960) | 0.756  (0.700–0.952) |
|  | LFM-Mix | 0.861  (0.805–0.915) | 0.755  (0.636–0.886) | 0.863  (0.722–0.963) | 0.877  (0.794–0.964) | 0.733  (0.635–0.852) |
|  | LFM-Seq | 0.920  (0.875–0.959) | 0.979  (0.824–1.000) | 0.740  (0.675–0.924) | 0.829  (0.780–0.941) | 0.964  (0.784–1.000) |
|  | FMCIB | 0.824  (0.755–0.883) | 0.681  (0.600–0.875) | 0.904  (0.685–0.969) | 0.901  (0.767–0.968) | 0.688  (0.610–0.823) |
|  | Radiomics | 0.750  (0.676–0.830) | 0.883  (0.745–0.964) | 0.562  (0.453–0.743) | 0.722  (0.646–0.823) | 0.788  (0.648–0.925) |

**Table S2. Performance of pathological grade assessment.**

| **Cohort** | **Model** | **AUC**  **(95% CI)** | **Sensitivity (95% CI)** | **Specificity (95% CI)** | **PPV**  **(95% CI)** | **NPV**  **(95% CI)** |
| --- | --- | --- | --- | --- | --- | --- |
| IVC | LFM-Base | 0.428  (0.332–0.523) | 0.932  (0.406–1.000) | 0.191  (0.078–0.746) | 0.553  (0.472–0.652) | 0.722  (0.550–1.000) |
|  | LFM-DL | 0.832  (0.762–0.895) | 0.685  (0.582–0.821) | 0.897  (0.797–0.971) | 0.877  (0.781–0.962) | 0.726  (0.646–0.831) |
|  | LFM-Mix | 0.792  (0.719–0.861) | 0.712  (0.575–0.867) | 0.779  (0.657–0.922) | 0.776  (0.684–0.901) | 0.716  (0.622–0.849) |
|  | LFM-Seq | 0.833  (0.758–0.896) | 0.836  (0.629–0.912) | 0.721  (0.623–0.923) | 0.762  (0.683–0.906) | 0.803  (0.654–0.899) |
|  | FMCIB | 0.517  (0.422–0.619) | 0.890  (0.493–1.000) | 0.265  (0.093–0.671) | 0.565  (0.482–0.676) | 0.692  (0.517–1.000) |
|  | Radiomics | 0.754  (0.663–0.834) | 0.534  (0.438–0.905) | 0.882  (0.526–0.967) | 0.830  (0.629–0.943) | 0.638  (0.580–0.860) |
| EVC1 | LFM-Base | 0.425  (0.337–0.517) | 0.018  (0.000–0.972) | 1.000  (0.061–1.000) | 1.000  (0.000–1.000) | 0.367  (0.311–0.615) |
|  | LFM-DL | 0.867  (0.806–0.925) | 0.890  (0.754–0.943) | 0.774  (0.705–0.915) | 0.874  (0.821–0.949) | 0.800  (0.646–0.893) |
|  | LFM-Mix | 0.832  (0.764–0.895) | 0.853  (0.699–0.917) | 0.758  (0.686–0.921) | 0.861  (0.811–0.944) | 0.746  (0.587–0.857) |
|  | LFM-Seq | 0.885  (0.826–0.935) | 0.899  (0.816–0.960) | 0.823  (0.727–0.922) | 0.899  (0.846–0.955) | 0.823  (0.703–0.923) |
|  | FMCIB | 0.496  (0.403–0.588) | 0.954  (0.171–0.991) | 0.210  (0.119–0.952) | 0.680  (0.608–0.867) | 0.722  (0.372–0.933) |
|  | Radiomics | 0.742  (0.664–0.818) | 0.606  (0.461–0.964) | 0.774  (0.400–0.911) | 0.825  (0.713–0.917) | 0.527  (0.448–0.857) |
| EVC2 | LFM-Base | 0.440  (0.353–0.524) | 0.034  (0.012–0.989) | 0.988  (0.037–1.000) | 0.750  (0.450–1.000) | 0.485  (0.431–0.910) |
|  | LFM-DL | 0.800  (0.732–0.867) | 0.816  (0.742–0.946) | 0.725  (0.568–0.824) | 0.763  (0.673–0.850) | 0.784  (0.707–0.923) |
|  | LFM-Mix | 0.775  (0.700–0.846) | 0.805  (0.629–0.905) | 0.688  (0.568–0.857) | 0.737  (0.655–0.851) | 0.764  (0.636–0.868) |
|  | LFM-Seq | 0.795  (0.724–0.860) | 0.897  (0.765–0.988) | 0.600  (0.481–0.779) | 0.709  (0.637–0.817) | 0.842  (0.706–0.975) |
|  | FMCIB | 0.451  (0.362–0.536) | 1.000  (0.162–1.000) | 0.087  (0.047–0.947) | 0.544  (0.472–0.792) | 1.000  (0.471–1.000) |
|  | Radiomics | 0.573  (0.487–0.661) | 0.954  (0.207–1.000) | 0.188  (0.133–0.939) | 0.561  (0.500–0.815) | 0.789  (0.479–1.000) |

**Table S3. Robustness analysis under tumor-localization perturbation for MVI assessment.**

| **Cohort** | **Model** | **5%** | **10%** | **15%** | **20%** | **25%** |
| --- | --- | --- | --- | --- | --- | --- |
| IVC | LFM-Base | 0.531  (0.523-0.538) | 0.461  (0.451-0.471) | 0.456  (0.446-0.465) | 0.455  (0.444-0.466) | 0.465  (0.452-0.478) |
|  | LFM-DL | 0.735  (0.728-0.741) | 0.733  (0.726-0.740) | 0.723  (0.715-0.732) | 0.713  (0.705-0.722) | 0.703  (0.693-0.712) |
|  | LFM-Mix | 0.872  (0.869-0.876) | 0.861  (0.858-0.865) | 0.854  (0.851-0.857) | 0.846  (0.841-0.852) | 0.829  (0.823-0.834) |
|  | LFM-Seq | 0.870  (0.865-0.874) | 0.870  (0.865-0.874) | 0.867  (0.862-0.873) | 0.854  (0.849-0.860) | 0.840  (0.833-0.846) |
|  | FMCIB | 0.694  (0.687-0.702) | 0.667  (0.657-0.677) | 0.628  (0.616-0.639) | 0.595  (0.583-0.607) | 0.556  (0.544-0.568) |
| EVC1 | LFM-Base | 0.496  (0.488-0.503) | 0.442  (0.435-0.450) | 0.437  (0.426-0.448) | 0.444  (0.434-0.455) | 0.460  (0.449-0.470) |
|  | LFM-DL | 0.731  (0.725-0.737) | 0.729  (0.723-0.735) | 0.716  (0.710-0.721) | 0.708  (0.702-0.714) | 0.711  (0.704-0.719) |
|  | LFM-Mix | 0.808  (0.805-0.811) | 0.807  (0.804-0.809) | 0.799  (0.796-0.803) | 0.796  (0.793-0.800) | 0.785  (0.781-0.789) |
|  | LFM-Seq | 0.787  (0.783-0.791) | 0.791  (0.786-0.795) | 0.787  (0.783-0.792) | 0.776  (0.770-0.781) | 0.766  (0.760-0.772) |
|  | FMCIB | 0.661  (0.653-0.670) | 0.644  (0.636-0.653) | 0.617  (0.607-0.627) | 0.576  (0.565-0.588) | 0.551 (0.541-0.562) |
| EVC2 | LFM-Base | 0.571  (0.566-0.576) | 0.516  (0.506-0.526) | 0.504  (0.494-0.514) | 0.520  (0.510-0.530) | 0.519  (0.510-0.529) |
|  | LFM-DL | 0.831  (0.827-0.836) | 0.826  (0.821-0.831) | 0.822  (0.817-0.828) | 0.815  (0.808-0.822) | 0.805  (0.798-0.811) |
|  | LFM-Mix | 0.867  (0.864-0.870) | 0.863  (0.860-0.866) | 0.855  (0.852-0.857) | 0.842  (0.838-0.846) | 0.828  (0.825-0.832) |
|  | LFM-Seq | 0.852  (0.847-0.857) | 0.852  (0.847-0.857) | 0.847  (0.842-0.851) | 0.840  (0.836-0.845) | 0.828  (0.822-0.834) |
|  | FMCIB | 0.634  (0.626-0.643) | 0.619  (0.610-0.628) | 0.583  (0.572-0.594) | 0.546  (0.534-0.559) | 0.526  (0.515-0.537) |

Note. Values are reported as mean AUC with 95% confidence interval in parentheses, calculated across 50 repeated runs. Perturbation was applied to the bounding-box coordinates of tumor regions. The performance of radiomics model was not evaluated as it depends on precise tumor boundary segmentation.

**Table S4. Robustness analysis under tumor-localization perturbation for pathological grade assessment.**

| **Cohort** | **Model** | **5%** | **10%** | **15%** | **20%** | **25%** |
| --- | --- | --- | --- | --- | --- | --- |
| IVC | LFM-Base | 0.565  (0.556-0.573) | 0.587  (0.578-0.596) | 0.581  (0.569-0.593) | 0.581  (0.572-0.589) | 0.560  (0.550-0.570) |
|  | LFM-DL | 0.796  (0.791-0.801) | 0.794  (0.789-0.800) | 0.783  (0.777-0.788) | 0.783  (0.777-0.789) | 0.761  (0.754-0.767) |
|  | LFM-Mix | 0.776  (0.771-0.781) | 0.772  (0.766-0.777) | 0.765  (0.761-0.770) | 0.752  (0.745-0.759) | 0.744  (0.736-0.751) |
|  | LFM-Seq | 0.782  (0.778-0.786) | 0.775  (0.770-0.780) | 0.762  (0.756-0.768) | 0.751  (0.745-0.757) | 0.737  (0.728-0.747) |
|  | FMCIB | 0.477  (0.471-0.484) | 0.471  (0.465-0.477) | 0.477  (0.468-0.485) | 0.482  (0.471-0.493) | 0.494  (0.485-0.504) |
| EVC1 | LFM-Base | 0.543  (0.535-0.552) | 0.552  (0.543-0.561) | 0.542  (0.532-0.551) | 0.514  (0.503-0.525) | 0.498  (0.487-0.509) |
|  | LFM-DL | 0.851  (0.849-0.853) | 0.856  (0.853-0.859) | 0.851  (0.848-0.855) | 0.846  (0.843-0.850) | 0.834  (0.830-0.839) |
|  | LFM-Mix | 0.823  (0.820-0.827) | 0.820  (0.817-0.823) | 0.813  (0.809-0.817) | 0.797  (0.792-0.801) | 0.785  (0.780-0.790) |
|  | LFM-Seq | 0.854 (0.850-0.858) | 0.847  (0.843-0.851) | 0.837  (0.832-0.842) | 0.815  (0.809-0.821) | 0.799  (0.792-0.806) |
|  | FMCIB | 0.449  (0.445-0.454) | 0.449  (0.441-0.457) | 0.470  (0.462-0.478) | 0.480  (0.469-0.490) | 0.490  (0.479-0.501) |
| EVC2 | LFM-Base | 0.510  (0.502-0.517) | 0.525  (0.516-0.533) | 0.521  (0.513-0.530) | 0.503  (0.494-0.512) | 0.497  (0.488-0.506) |
|  | LFM-DL | 0.774  (0.771-0.778) | 0.770  (0.766-0.774) | 0.766  (0.762-0.770) | 0.751  (0.747-0.756) | 0.747  (0.741-0.754) |
|  | LFM-Mix | 0.771  (0.767-0.775) | 0.765  (0.760-0.769) | 0.751  (0.747-0.755) | 0.739  (0.734-0.744) | 0.729  (0.725-0.734) |
|  | LFM-Seq | 0.775  (0.772-0.778) | 0.772  (0.766-0.777) | 0.756  (0.751-0.762) | 0.732  (0.726-0.739) | 0.715  (0.709-0.722) |
|  | FMCIB | 0.437  (0.430-0.444) | 0.445  (0.437-0.453) | 0.439  (0.429-0.449) | 0.466  (0.457-0.475) | 0.474  (0.464-0.484) |

Note. Values are reported as mean AUC with 95% confidence interval in parentheses, calculated across 50 repeated runs. Perturbation was applied to the bounding-box coordinates of tumor regions. The performance of radiomics model was not evaluated as it depends on precise tumor boundary segmentation.

**Table S5. Performance with limited training data size for MVI assessment.**

| **Cohort** | **Model** | **10%** | **20%** | **50%** |
| --- | --- | --- | --- | --- |
| IVC | LFM-Base | 0.624 (0.610-0.637) | 0.630 (0.618-0.642) | 0.633 (0.620-0.646) |
|  | LFM-DL | 0.663 (0.635-0.692) | 0.748 (0.722-0.774) | 0.828 (0.810-0.847) |
|  | LFM-Mix | 0.674 (0.650-0.698) | 0.756 (0.743-0.769) | 0.810 (0.797-0.824) |
|  | LFM-Seq | 0.663 (0.634-0.692) | 0.748 (0.722-0.774) | 0.829 (0.810-0.847) |
|  | FMCIB | 0.639 (0.621-0.658) | 0.612 (0.596-0.629) | 0.652 (0.635-0.669) |
|  | Radiomics | 0.631 (0.595-0.666) | 0.730 (0.698-0.763) | 0.803 (0.784-0.822) |
| EVC1 | LFM-Base | 0.572 (0.560-0.585) | 0.579 (0.567-0.591) | 0.585 (0.572-0.598) |
|  | LFM-DL | 0.629 (0.604-0.655) | 0.702 (0.680-0.723) | 0.766 (0.751-0.780) |
|  | LFM-Mix | 0.655 (0.632-0.679) | 0.727 (0.713-0.740) | 0.770 (0.758-0.782) |
|  | LFM-Seq | 0.630 (0.605-0.656) | 0.702 (0.681-0.724) | 0.766 (0.752-0.781) |
|  | FMCIB | 0.592 (0.577-0.608) | 0.568 (0.554-0.582) | 0.629 (0.613-0.645) |
|  | Radiomics | 0.630 (0.594-0.667) | 0.717 (0.687-0.747) | 0.783 (0.767-0.800) |
| EVC2 | LFM-Base | 0.616 (0.600-0.631) | 0.611 (0.592-0.629) | 0.615 (0.599-0.632) |
|  | LFM-DL | 0.664 (0.634-0.693) | 0.742 (0.715-0.770) | 0.822 (0.802-0.842) |
|  | LFM-Mix | 0.674 (0.648-0.700) | 0.751 (0.734-0.768) | 0.805 (0.789-0.821) |
|  | LFM-Seq | 0.664 (0.634-0.693) | 0.744 (0.717-0.771) | 0.823 (0.803-0.843) |
|  | FMCIB | 0.595 (0.576-0.613) | 0.586 (0.569-0.602) | 0.610 (0.595-0.625) |
|  | Radiomics | 0.626 (0.589-0.664) | 0.721 (0.688-0.755) | 0.788 (0.769-0.807) |

Note. Values are reported as mean AUC with 95% confidence interval in parentheses, calculated across 50 repeated runs using stratified random sampling of 10%, 20%, and 50% of the training data.

**Table S6. Performance with limited training data size for pathological grade assessment.**

| **Cohort** | **Model** | **10%** | **20%** | **50%** |
| --- | --- | --- | --- | --- |
| IVC | LFM-Base | 0.566 (0.556-0.575) | 0.566 (0.556-0.575) | 0.546 (0.535-0.557) |
|  | LFM-DL | 0.635 (0.610-0.660) | 0.688 (0.666-0.710) | 0.767 (0.753-0.781) |
|  | LFM-Mix | 0.627 (0.602-0.653) | 0.676 (0.652-0.700) | 0.746 (0.733-0.760) |
|  | LFM-Seq | 0.635 (0.611-0.660) | 0.688 (0.666-0.710) | 0.767 (0.753-0.781) |
|  | FMCIB | 0.622 (0.609-0.635) | 0.607 (0.595-0.620) | 0.598 (0.584-0.613) |
|  | Radiomics | 0.552 (0.523-0.580) | 0.593 (0.569-0.617) | 0.666 (0.650-0.683) |
| EVC1 | LFM-Base | 0.540 (0.530-0.549) | 0.539 (0.531-0.546) | 0.529 (0.519-0.538) |
|  | LFM-DL | 0.649 (0.618-0.681) | 0.720 (0.689-0.750) | 0.809 (0.794-0.823) |
|  | LFM-Mix | 0.636 (0.602-0.670) | 0.713 (0.682-0.743) | 0.806 (0.791-0.820) |
|  | LFM-Seq | 0.649 (0.618-0.680) | 0.718 (0.687-0.748) | 0.808 (0.793-0.823) |
|  | FMCIB | 0.610 (0.594-0.627) | 0.601 (0.580-0.621) | 0.593 (0.572-0.614) |
|  | Radiomics | 0.558 (0.522-0.593) | 0.599 (0.569-0.630) | 0.667 (0.639-0.695) |
| EVC2 | LFM-Base | 0.530 (0.523-0.537) | 0.530 (0.524-0.536) | 0.522 (0.515-0.529) |
|  | LFM-DL | 0.602 (0.580-0.624) | 0.651 (0.630-0.673) | 0.726 (0.713-0.739) |
|  | LFM-Mix | 0.609 (0.583-0.636) | 0.668 (0.645-0.691) | 0.748 (0.737-0.759) |
|  | LFM-Seq | 0.602 (0.581-0.623) | 0.649 (0.628-0.671) | 0.726 (0.713-0.739) |
|  | FMCIB | 0.545 (0.534-0.557) | 0.546 (0.535-0.558) | 0.548 (0.536-0.561) |
|  | Radiomics | 0.551 (0.522-0.580) | 0.593 (0.567-0.619) | 0.637 (0.614-0.660) |

Note. Values are reported as mean AUC with 95% confidence interval in parentheses, calculated across 50 repeated runs using stratified random sampling of 10%, 20%, and 50% of the training data.

**Table S7. Tumor size subgroup analysis for MVI assessment.**

| **Subgroup** | **Model** | **IVC** | **EVC1** | **EVC2** |
| --- | --- | --- | --- | --- |
| <=3 cm  (IVC: n=43, MVI+ 1;  EVC1: n=33, MVI+ 3;  EVC2: n=23, MVI+ 4) | LFM-Base | 0.309  (0.170-0.464) | 0.561  (0.219-0.938) | 0.579  (0.167-0.900) |
|  | LFM-DL | 0.667  (0.537-0.790) | 0.878  (0.710-0.982) | 0.868  (0.708-1.000) |
|  | LFM-Mix | 0.738  (0.595-0.857) | 0.578  (0.000-0.952) | 0.618  (0.100-0.917) |
|  | LFM-Seq | 1.000  (1.000-1.000) | 0.900  (0.774-1.000) | 0.947  (0.828-1.000) |
|  | FMCIB | 0.405  (0.262-0.529) | 0.439  (0.167-0.696) | 0.474  (0.091-0.809) |
|  | Radiomics | 0.714  (0.582-0.851) | 0.844  (0.656-0.969) | 0.697  (0.429-0.909) |
| 3-5 cm  (IVC: n=40, MVI+ 8;  EVC1: n=43, MVI+ 7;  EVC2: n=41, MVI+ 12) | LFM-Base | 0.422  (0.208-0.654) | 0.248  (0.043-0.502) | 0.484  (0.281-0.699) |
|  | LFM-DL | 0.859  (0.686-0.985) | 0.786  (0.562-0.937) | 0.954  (0.889-1.000) |
|  | LFM-Mix | 0.715  (0.417-0.975) | 0.595  (0.300-0.870) | 0.744  (0.557-0.904) |
|  | LFM-Seq | 0.871  (0.759-0.969) | 0.905  (0.804-0.990) | 0.974  (0.927-1.000) |
|  | FMCIB | 0.648  (0.460-0.829) | 0.675  (0.426-0.887) | 0.744  (0.540-0.909) |
|  | Radiomics | 0.613  (0.405-0.789) | 0.718  (0.498-0.906) | 0.664  (0.448-0.866) |
| >5 cm  (IVC: n=58, MVI+ 35;  EVC1: n=95, MVI+ 65;  EVC2: n=103, MVI+ 78) | LFM-Base | 0.600  (0.438-0.759) | 0.601  (0.479-0.732) | 0.586  (0.468-0.719) |
|  | LFM-DL | 0.805  (0.674-0.906) | 0.705  (0.584-0.836) | 0.827  (0.724-0.918) |
|  | LFM-Mix | 0.711  (0.562-0.848) | 0.682  (0.570-0.805) | 0.815  (0.703-0.908) |
|  | LFM-Seq | 0.784  (0.641-0.901) | 0.769  (0.647-0.893) | 0.844  (0.729-0.934) |
|  | FMCIB | 0.649  (0.500-0.779) | 0.764  (0.657-0.880) | 0.782  (0.665-0.876) |
|  | Radiomics | 0.646  (0.486-0.785) | 0.736  (0.608-0.845) | 0.604  (0.468-0.734) |

Note. Values are reported as AUC (95% CI). AUC = area under the receiver operating characteristic curve; CI = confidence interval.

**Table S8. Tumor size subgroup analysis for pathological grade assessment.**

| **Subgroup** | **Model** | **IVC** | **EVC1** | **EVC2** |
| --- | --- | --- | --- | --- |
| <=3 cm  (IVC: n=43, high-grade 12;  EVC1: n=33, high-grade 11;  EVC2: n=23, high-grade 4) | LFM-Base | 0.446  (0.255-0.610) | 0.572  (0.342-0.780) | 0.868  (0.633-1.000) |
|  | LFM-DL | 0.629  (0.430-0.797) | 0.769  (0.561-0.924) | 0.803  (0.430-1.000) |
|  | LFM-Mix | 0.633  (0.451-0.804) | 0.711  (0.461-0.900) | 0.684  (0.237-1.000) |
|  | LFM-Seq | 0.645  (0.463-0.829) | 0.793  (0.602-0.937) | 0.908  (0.740-1.000) |
|  | FMCIB | 0.583  (0.377-0.769) | 0.653  (0.422-0.859) | 0.842  (0.553-1.000) |
|  | Radiomics | 0.634  (0.429-0.825) | 0.636  (0.423-0.820) | 0.697  (0.500-0.900) |
| 3-5 cm  (IVC: n=40, high-grade 19;  EVC1: n=43, high-grade 15;  EVC2: n=41, high-grade 12) | LFM-Base | 0.608  (0.451-0.792) | 0.579  (0.399-0.754) | 0.476  (0.288-0.648) |
|  | LFM-DL | 0.777  (0.623-0.911) | 0.845  (0.709-0.966) | 0.862  (0.739-0.957) |
|  | LFM-Mix | 0.754  (0.578-0.892) | 0.762  (0.558-0.918) | 0.678  (0.464-0.869) |
|  | LFM-Seq | 0.794  (0.644-0.934) | 0.890  (0.782-0.980) | 0.800  (0.649-0.917) |
|  | FMCIB | 0.561  (0.386-0.724) | 0.670  (0.500-0.822) | 0.466  (0.273-0.652) |
|  | Radiomics | 0.561  (0.374-0.738) | 0.624  (0.461-0.786) | 0.534  (0.335-0.726) |
| >5 cm  (IVC: n=58, high-grade 42;  EVC1: n=95, high-grade 83;  EVC2: n=103, high-grade 71) | LFM-Base | 0.467  (0.254-0.641) | 0.617  (0.477-0.753) | 0.518  (0.412-0.637) |
|  | LFM-DL | 0.884  (0.777-0.980) | 0.680  (0.490-0.876) | 0.653  (0.515-0.771) |
|  | LFM-Mix | 0.750  (0.590-0.891) | 0.581  (0.395-0.782) | 0.645  (0.501-0.770) |
|  | LFM-Seq | 0.848  (0.720-0.952) | 0.747  (0.578-0.904) | 0.674  (0.532-0.786) |
|  | FMCIB | 0.423  (0.235-0.605) | 0.436  (0.236-0.649) | 0.481  (0.367-0.594) |
|  | Radiomics | 0.731  (0.582-0.869) | 0.740  (0.578-0.874) | 0.392  (0.283-0.500) |

Note. Values are reported as AUC (95% CI). AUC = area under the receiver operating characteristic curve; CI = confidence interval.

**Table S9. RFS prediction performance of models.**

| **Cohort** | **Model** | **C-index** | **12-month AUC** | **24-month AUC** | **36-month AUC** |
| --- | --- | --- | --- | --- | --- |
| EVC1 | Clinical | 0.641  (0.585-0.690) | 0.690  (0.596-0.766) | 0.656  (0.551-0.754) | 0.669  (0.528-0.795) |
|  | Clinicopathological | 0.677  (0.622-0.727) | 0.733  (0.651-0.807) | 0.722  (0.630-0.802) | 0.747  (0.646-0.852) |
|  | LFM-Base | 0.702  (0.646-0.748) | 0.728  (0.639-0.809) | 0.835  (0.769-0.899) | 0.853  (0.758-0.916) |
|  | LFM-DL | 0.727  (0.676-0.772) | 0.771  (0.699-0.835) | 0.834  (0.743-0.908) | 0.915  (0.854-0.962) |
|  | LFM-Mix | 0.723  (0.673-0.770) | 0.774  (0.689-0.841) | 0.837  (0.747-0.910) | 0.891  (0.800-0.952) |
|  | LFM-Seq | 0.728  (0.674-0.778) | 0.786  (0.715-0.853) | 0.832  (0.754-0.910) | 0.897  (0.830-0.950) |
|  | FMCIB | 0.694  (0.643-0.744) | 0.780  (0.704-0.841) | 0.723  (0.609-0.825) | 0.753  (0.611-0.869) |
|  | Radiomics | 0.701  (0.644-0.751) | 0.751  (0.674-0.822) | 0.797  (0.714-0.874) | 0.919  (0.866-0.962) |
| EVC2 | Clinical | 0.649  (0.591-0.703) | 0.678  (0.590-0.758) | 0.651  (0.556-0.731) | 0.626  (0.506-0.728) |
|  | Clinicopathological | 0.671  (0.625-0.723) | 0.732  (0.655-0.805) | 0.702  (0.617-0.787) | 0.663  (0.554-0.765) |
|  | LFM-Base | 0.677  (0.615-0.734) | 0.734  (0.646-0.814) | 0.710  (0.626-0.793) | 0.695  (0.594-0.793) |
|  | LFM-DL | 0.697  (0.645-0.750) | 0.759  (0.678-0.833) | 0.714  (0.636-0.799) | 0.702  (0.597-0.805) |
|  | LFM-Mix | 0.704  (0.646-0.759) | 0.768  (0.686-0.837) | 0.721  (0.642-0.800) | 0.712  (0.599-0.817) |
|  | LFM-Seq | 0.701  (0.647-0.755) | 0.776  (0.698-0.844) | 0.720  (0.633-0.797) | 0.696  (0.587-0.808) |
|  | FMCIB | 0.625  (0.566-0.689) | 0.689  (0.598-0.777) | 0.629  (0.525-0.720) | 0.578  (0.451-0.684) |
|  | Radiomics | 0.636  (0.570-0.699) | 0.680  (0.591-0.761) | 0.657  (0.562-0.745) | 0.678  (0.566-0.781) |

Note. Values are reported as C-index with 95% confidence interval in parentheses. C-index = Harrell's concordance index; AUC = area under the receiver operating characteristic curve; CI = confidence interval.

**Table S10. Univariable and multivariable Cox regression analyses for RFS prediction.**

| Variable | EVC1 | | | |  | EVC2 | | | |
| --- | --- | --- | --- | --- | --- | --- | --- | --- | --- |
|  | Univariable HR (95% CI) | P value | Multivariable HR (95% CI) | P  value |  | Univariable HR (95% CI) | P  value | Multivariable HR (95% CI) | P  value |
| AFP  (>200 ng/mL) | 1.605  (1.128-2.285) | 0.009 | 0.996  (0.674-1.473) | 0.985 |  | 1.825  (1.244-2.677) | 0.002 | 1.368  (0.901-2.076) | 0.142 |
| ALT  (>50 IU/L) | 1.140  (0.746-1.744) | 0.545 | 0.928  (0.556-1.549) | 0.775 |  | 1.048  (0.647-1.696) | 0.849 | 1.040  (0.583-1.855) | 0.896 |
| AST  (>40 IU/L) | 1.501  (1.061-2.123) | 0.022 | 1.155  (0.749-1.781) | 0.515 |  | 1.356  (0.925-1.987) | 0.118 | 0.943  (0.598-1.489) | 0.803 |
| Age  (per year) | 1.007  (0.992-1.021) | 0.381 | 1.006  (0.990-1.022) | 0.465 |  | 1.003  (0.987-1.019) | 0.740 | 1.009  (0.992-1.026) | 0.291 |
| GGT  (>60 IU/L) | 1.406  (0.995-1.988) | 0.053 | 0.772  (0.501-1.190) | 0.241 |  | 1.645  (1.108-2.441) | 0.013 | 1.402  (0.914-2.148) | 0.121 |
| HBsAg  (>=0.05 IU/mL) | 1.001  (0.651-1.539) | 0.997 | 1.405  (0.862-2.288) | 0.173 |  | 0.985  (0.593-1.637) | 0.953 | 1.185  (0.696-2.018) | 0.532 |
| LFM-Seq risk score  (per 1 SD) | 2.525  (2.026-3.146) | <0.001 | 2.896  (2.112-3.971) | <0.001 |  | 1.945  (1.527-2.477) | <0.001 | 1.657  (1.148-2.392) | 0.007 |
| MVI (positive) | 2.281  (1.602-3.248) | <0.001 | 1.095  (0.718-1.670) | 0.675 |  | 2.135  (1.431-3.183) | <0.001 | 1.126  (0.673-1.885) | 0.651 |
| Pathological grade  (high-grade) | 2.021  (1.388-2.941) | <0.001 | 0.913  (0.579-1.440) | 0.696 |  | 2.024  (1.364-3.002) | <0.001 | 1.305  (0.824-2.067) | 0.256 |
| Sex  (male) | 0.910  (0.553-1.498) | 0.711 | 1.384  (0.794-2.412) | 0.252 |  | 1.019  (0.597-1.737) | 0.946 | 0.851  (0.489-1.482) | 0.569 |
| Tumor size  (>3 cm) | 1.654  (1.044-2.619) | 0.032 | 0.723  (0.426-1.227) | 0.229 |  | 1.845  (0.986-3.452) | 0.055 | 0.867  (0.420-1.792) | 0.701 |

Note. HR = hazard ratio; CI = confidence interval. The HR for the LFM-Seq risk score are per 1 SD increment, using EVC1 as the reference for standardization. The multivariable model included all variables listed in the table.

**Table S11. Tumor size subgroup analysis for C-index of RFS prediction.**

| **Subgroup** | **Model** | **EVC1** | **EVC2** |
| --- | --- | --- | --- |
| <=3 cm | Clinical | 0.546  (0.394-0.687) | 0.538  (0.307-0.779) |
|  | Clinicopathological | 0.560  (0.427-0.707) | 0.543  (0.288-0.766) |
|  | LFM-Base | 0.699  (0.570-0.803) | 0.762  (0.609-0.895) |
|  | LFM-DL | 0.657  (0.519-0.798) | 0.715  (0.528-0.867) |
|  | LFM-Mix | 0.623  (0.474-0.759) | 0.683  (0.500-0.827) |
|  | LFM-Seq | 0.658  (0.509-0.799) | 0.751  (0.567-0.891) |
|  | FMCIB | 0.726  (0.564-0.849) | 0.588  (0.359-0.808) |
|  | Radiomics | 0.618  (0.467-0.739) | 0.609  (0.399-0.796) |
| 3-5 cm | Clinical | 0.508  (0.387-0.611) | 0.464  (0.330-0.594) |
|  | Clinicopathological | 0.574  (0.441-0.694) | 0.607  (0.477-0.733) |
|  | LFM-Base | 0.731  (0.633-0.822) | 0.730  (0.594-0.856) |
|  | LFM-DL | 0.710  (0.598-0.804) | 0.691  (0.578-0.793) |
|  | LFM-Mix | 0.715  (0.604-0.819) | 0.707  (0.585-0.813) |
|  | LFM-Seq | 0.680  (0.557-0.791) | 0.732  (0.630-0.831) |
|  | FMCIB | 0.596  (0.462-0.716) | 0.477  (0.330-0.601) |
|  | Radiomics | 0.702  (0.576-0.798) | 0.643  (0.512-0.775) |
| >5 cm | Clinical | 0.638  (0.563-0.707) | 0.638  (0.573-0.703) |
|  | Clinicopathological | 0.620  (0.541-0.697) | 0.634  (0.556-0.714) |
|  | LFM-Base | 0.641  (0.565-0.710) | 0.611  (0.530-0.695) |
|  | LFM-DL | 0.669  (0.601-0.739) | 0.663  (0.584-0.731) |
|  | LFM-Mix | 0.673  (0.588-0.744) | 0.683  (0.607-0.752) |
|  | LFM-Seq | 0.689  (0.610-0.763) | 0.649  (0.580-0.722) |
|  | FMCIB | 0.627  (0.551-0.704) | 0.610  (0.524-0.682) |
|  | Radiomics | 0.639  (0.555-0.715) | 0.592  (0.508-0.670) |

Note. Values are reported as C-index with 95% confidence interval in parentheses. C-index = Harrell's concordance index; CI = confidence interval.

**Table S12. Tumor size subgroup analysis for time-dependent AUC of RFS prediction.**

| **Subgroup** | **Model** | **12-month AUC** | | **24-month AUC** | | **36-month AUC** | |
| --- | --- | --- | --- | --- | --- | --- | --- |
|  |  | **EVC1** | **EVC2** | **EVC1** | **EVC2** | **EVC1** | **EVC2** |
| <=3 cm | Clinical | 0.659  (0.420-0.887) | 0.462  (0.231-0.686) | 0.425  (0.188-0.680) | 0.784  (0.447-1.000) | 0.503  (0.245-0.842) | 0.365  (0.125-0.662) |
|  | Clinicopathological | 0.513  (0.251-0.770) | 0.708  (0.228-1.000) | 0.664  (0.460-0.856) | 0.566  (0.254-0.885) | 0.604  (0.371-0.817) | 0.421  (0.128-0.727) |
|  | LFM-Base | 0.635  (0.405-0.837) | 0.867  (0.658-1.000) | 0.819  (0.616-0.947) | 0.798  (0.546-1.000) | 0.800  (0.594-0.951) | 0.825  (0.616-1.000) |
|  | LFM-DL | 0.582  (0.323-0.807) | 0.850  (0.602-1.000) | 0.866  (0.713-0.991) | 0.774  (0.525-0.957) | 0.800  (0.556-0.949) | 0.762  (0.461-0.975) |
|  | LFM-Mix | 0.481  (0.244-0.706) | 0.717  (0.468-0.932) | 0.784  (0.517-0.961) | 0.774  (0.556-1.000) | 0.850  (0.618-1.000) | 0.873  (0.631-1.000) |
|  | LFM-Seq | 0.603  (0.370-0.827) | 0.883  (0.686-1.000) | 0.889  (0.749-0.987) | 0.893  (0.671-1.000) | 0.825  (0.627-0.957) | 0.794  (0.515-1.000) |
|  | FMCIB | 0.799  (0.575-0.989) | 0.667  (0.353-1.000) | 0.807  (0.592-0.975) | 0.679  (0.391-0.917) | 0.733  (0.411-0.973) | 0.619  (0.294-0.890) |
|  | Radiomics | 0.561  (0.342-0.767) | 0.700  (0.407-0.940) | 0.778  (0.536-0.963) | 0.619  (0.322-0.886) | 0.783  (0.532-0.950) | 0.603  (0.264-0.882) |
| 3-5 cm | Clinical | 0.540  (0.329-0.744) | 0.562  (0.405-0.734) | 0.562  (0.368-0.774) | 0.457  (0.250-0.704) | 0.382  (0.202-0.571) | 0.471  (0.180-0.706) |
|  | Clinicopathological | 0.659  (0.451-0.846) | 0.653  (0.393-0.864) | 0.561  (0.366-0.742) | 0.622  (0.422-0.806) | 0.585  (0.354-0.797) | 0.611  (0.357-0.823) |
|  | LFM-Base | 0.731  (0.559-0.898) | 0.806  (0.578-0.992) | 0.880  (0.767-0.973) | 0.763  (0.590-0.912) | 0.804  (0.638-0.932) | 0.710  (0.500-0.905) |
|  | LFM-DL | 0.669  (0.480-0.818) | 0.853  (0.679-0.965) | 0.832  (0.682-0.942) | 0.744  (0.566-0.893) | 0.881  (0.744-0.968) | 0.727  (0.533-0.893) |
|  | LFM-Mix | 0.701  (0.500-0.875) | 0.823  (0.667-0.948) | 0.842  (0.702-0.951) | 0.733  (0.537-0.893) | 0.804  (0.629-0.930) | 0.784  (0.613-0.925) |
|  | LFM-Seq | 0.640  (0.432-0.831) | 0.845  (0.704-0.948) | 0.815  (0.639-0.933) | 0.719  (0.508-0.893) | 0.811  (0.621-0.938) | 0.795  (0.619-0.947) |
|  | FMCIB | 0.718  (0.515-0.897) | 0.560  (0.308-0.792) | 0.595  (0.391-0.784) | 0.400  (0.199-0.615) | 0.552  (0.312-0.779) | 0.455  (0.212-0.693) |
|  | Radiomics | 0.718  (0.504-0.890) | 0.659  (0.441-0.847) | 0.783  (0.605-0.934) | 0.719  (0.522-0.907) | 0.848  (0.705-0.967) | 0.824  (0.633-0.974) |
| >5 cm | Clinical | 0.614  (0.470-0.730) | 0.653  (0.437-0.864) | 0.619  (0.097-0.953) | 0.634  (0.525-0.746) | 0.661  (0.541-0.768) | 0.651  (0.496-0.770) |
|  | Clinicopathological | 0.611  (0.493-0.726) | 0.656  (0.540-0.777) | 0.704  (0.543-0.857) | 0.666  (0.532-0.793) | 0.535  (0.116-0.920) | 0.668  (0.504-0.806) |
|  | LFM-Base | 0.663  (0.542-0.781) | 0.633  (0.520-0.750) | 0.727  (0.551-0.891) | 0.623  (0.499-0.758) | 0.788  (0.475-0.961) | 0.652  (0.507-0.811) |
|  | LFM-DL | 0.723  (0.605-0.833) | 0.670  (0.568-0.773) | 0.759  (0.589-0.911) | 0.646  (0.527-0.761) | 0.948  (0.838-1.000) | 0.671  (0.544-0.788) |
|  | LFM-Mix | 0.759  (0.653-0.855) | 0.713  (0.602-0.813) | 0.799  (0.641-0.930) | 0.660  (0.541-0.775) | 0.844  (0.649-1.000) | 0.665  (0.527-0.808) |
|  | LFM-Seq | 0.781  (0.675-0.874) | 0.700  (0.591-0.799) | 0.761  (0.601-0.914) | 0.627  (0.508-0.749) | 0.918  (0.823-1.000) | 0.623  (0.476-0.768) |
|  | FMCIB | 0.693  (0.568-0.806) | 0.651  (0.536-0.772) | 0.614  (0.387-0.843) | 0.609  (0.473-0.755) | 0.766  (0.250-1.000) | 0.540  (0.366-0.721) |
|  | Radiomics | 0.669  (0.552-0.779) | 0.594  (0.482-0.700) | 0.709  (0.540-0.865) | 0.545  (0.405-0.682) | 0.978  (0.921-1.000) | 0.587  (0.423-0.742) |

**Table S13. Time-dependent hazard ratios of the LFM-Seq risk score.**

| Cohort | Model | 6 months | 12 months | 24 months | 36 months |
| --- | --- | --- | --- | --- | --- |
| EVC1 | Univariable | 2.68  (2.098-3.414) | 2.41  (1.903-3.043) | 2.16  (1.548-3.024) | 2.03  (1.341-3.085) |
|  | Multivariable | 3.02  (2.182-4.175) | 2.70  (1.928-3.769) | 2.41  (1.561-3.715) | 2.25  (1.348-3.769) |
| EVC2 | Univariable | 2.56  (1.907-3.422) | 1.78  (1.384-2.287) | 1.24  (0.894-1.716) | 1.00  (0.670-1.498) |
|  | Multivariable | 2.14  (1.430-3.214) | 1.52  (1.037-2.219) | 1.07  (0.690-1.670) | 0.88  (0.527-1.457) |

Note. Time-dependent hazard ratio (HR) was estimated from Cox models with a log-time interaction term tt(x) = x × log(t/12). Time-interaction P values: EVC1 univariable P = 0.234, multivariable P = 0.232; EVC2 univariable P < 0.001, multivariable P < 0.001. HRs are per 1 standard deviation increment of the risk score, using EVC1 as the reference for standardization.

**Table S14. Incremental value of the LFM-Seq risk score over the clinical model.**

| **Cohort** | **Time (months)** | **IDI (95% CI)** | **IDI P value** | **NRI (95% CI)** | **NRI P value** |
| --- | --- | --- | --- | --- | --- |
| EVC1 | 6 | 0.162  (0.081-0.241) | <0.001 | 0.303  (0.123-0.480) | 0.004 |
|  | 12 | 0.203  (0.117-0.288) | <0.001 | 0.341  (0.188-0.490) | <0.001 |
|  | 24 | 0.216  (0.127-0.321) | <0.001 | 0.397  (0.224-0.574) | <0.001 |
|  | 36 | 0.221  (0.118-0.332) | <0.001 | 0.584  (0.337-0.704) | <0.001 |
| EVC2 | 6 | 0.073  (0.024-0.148) | <0.001 | 0.342  (0.073-0.463) | 0.004 |
|  | 12 | 0.092  (0.037-0.178) | <0.001 | 0.285  (0.067-0.421) | 0.004 |
|  | 24 | 0.083  (0.022-0.177) | 0.016 | 0.244  (0.033-0.413) | 0.024 |
|  | 36 | 0.074  (0.006-0.169) | 0.020 | 0.197  (-0.012-0.382) | 0.064 |

Note. IDI = integrated discrimination improvement; NRI = net reclassification improvement (continuous). The reference model is the clinical model; the fusion model adds the LFM-Seq risk score; CI = confidence interval.

**Table S15. Calibration metrics for MVI assessment.**

| **Model** | **Cohort** | **Brier score** | **Calibration intercept** | **Calibration slope** |
| --- | --- | --- | --- | --- |
| LFM-Base | IVC | 0.204 | 0.250 | 1.520 |
|  | EVC1 | 0.251 | 0.508 | 1.079 |
|  | EVC2 | 0.293 | 1.588 | 1.781 |
| LFM-DL | IVC | 0.132 | 0.069 | 1.954 |
|  | EVC1 | 0.173 | 0.192 | 1.395 |
|  | EVC2 | 0.175 | 1.553 | 2.731 |
| LFM-Mix | IVC | 0.152 | 0.284 | 1.784 |
|  | EVC1 | 0.191 | 0.384 | 1.423 |
|  | EVC2 | 0.204 | 1.657 | 2.501 |
| LFM-Seq | IVC | 0.120 | -0.054 | 1.760 |
|  | EVC1 | 0.167 | 0.207 | 1.254 |
|  | EVC2 | 0.168 | 1.796 | 2.685 |
| FMCIB | IVC | 0.197 | 0.896 | 2.468 |
|  | EVC1 | 0.231 | 1.979 | 3.704 |
|  | EVC2 | 0.265 | 2.771 | 4.287 |
| Radiomics | IVC | 0.173 | 0.050 | 1.374 |
|  | EVC1 | 0.194 | 0.741 | 2.095 |
|  | EVC2 | 0.226 | 0.535 | 0.925 |

**Table S16. Calibration metrics for pathological grade assessment.**

| **Model** | **Cohort** | **Brier score** | **Calibration intercept** | **Calibration slope** |
| --- | --- | --- | --- | --- |
| LFM-Base | IVC | 0.255 | 0.051 | -0.676 |
|  | EVC1 | 0.257 | 0.510 | -2.134 |
|  | EVC2 | 0.254 | 0.053 | -1.750 |
| LFM-DL | IVC | 0.165 | -0.095 | 1.321 |
|  | EVC1 | 0.142 | 0.290 | 1.274 |
|  | EVC2 | 0.185 | -0.455 | 1.179 |
| LFM-Mix | IVC | 0.192 | 0.011 | 1.641 |
|  | EVC1 | 0.167 | 0.148 | 1.708 |
|  | EVC2 | 0.207 | -0.590 | 1.508 |
| LFM-Seq | IVC | 0.166 | -0.030 | 1.025 |
|  | EVC1 | 0.129 | 0.267 | 1.211 |
|  | EVC2 | 0.191 | -0.662 | 1.095 |
| FMCIB | IVC | 0.252 | 0.102 | 0.403 |
|  | EVC1 | 0.255 | 0.566 | 0.110 |
|  | EVC2 | 0.256 | 0.087 | -0.444 |
| Radiomics | IVC | 0.204 | 0.075 | 1.262 |
|  | EVC1 | 0.195 | 0.148 | 1.596 |
|  | EVC2 | 0.267 | -0.050 | 0.148 |

**Table S17. Ablation results for MVI assessment.**

| **Cohort** | **Model variant** | **AUC (95% CI)** | **DeLong *P* (vs LFM-Seq)** |
| --- | --- | --- | --- |
| IVC | LFM-Seq | 0.916 (0.870–0.957) | — |
|  | AP_only | 0.894 (0.839-0.949) | 0.151 |
|  | PV_only | 0.892 (0.834-0.950) | 0.174 |
|  | simple_fusion | 0.880 (0.823-0.937) | 0.017 |
|  | mean_pool | 0.912 (0.865-0.960) | 0.683 |
|  | max_pool | 0.881 (0.824-0.937) | 0.023 |
| EVC1 | LFM-Seq | 0.846 (0.784–0.900) | — |
|  | AP_only | 0.816 (0.754-0.878) | 0.040 |
|  | PV_only | 0.814 (0.750-0.878) | 0.040 |
|  | simple_fusion | 0.821 (0.758-0.885) | 0.070 |
|  | mean_pool | 0.818 (0.756-0.880) | 0.049 |
|  | max_pool | 0.822 (0.760-0.883) | 0.087 |
| EVC2 | LFM-Seq | 0.920 (0.875–0.959) | — |
|  | AP_only | 0.877 (0.822-0.932) | 0.014 |
|  | PV_only | 0.875 (0.822-0.928) | 0.005 |
|  | simple_fusion | 0.893 (0.843-0.943) | 0.053 |
|  | mean_pool | 0.869 (0.815-0.924) | 0.002 |
|  | max_pool | 0.849 (0.790-0.909) | <0.001 |

Note. All ablations were performed with the LFM-Seq. The AP_only and PV_only use a single imaging phase, AP_only refers to arterial-phase input only and PV_only refers to portal venous-phase input only; simple fusion replaces cross-phase attention with direct concatenation; mean_pool and max_pool replace attention-based multiple instance learning (ABMIL) with mean or max pooling of patch features. AUCs were computed on the best checkpoint per task; 95% CIs and P values were obtained with the DeLong method, comparing each variant with the LFM-Seq model on the same cohort.

**Table S18. Ablation results for pathological grade assessment.**

| **Cohort** | **Model variant** | **AUC (95% CI)** | **DeLong P (vs LFM-Seq)** |
| --- | --- | --- | --- |
| IVC | LFM-Seq | 0.833 (0.758–0.896) | — |
|  | AP_only | 0.804 (0.731-0.878) | 0.309 |
|  | PV_only | 0.796 (0.722-0.870) | 0.086 |
|  | simple_fusion | 0.809 (0.738-0.879) | 0.234 |
|  | mean_pool | 0.813 (0.742-0.885) | 0.320 |
|  | max_pool | 0.824 (0.756-0.892) | 0.621 |
| EVC1 | LFM-Seq | 0.885 (0.826–0.935) | — |
|  | AP_only | 0.802 (0.732-0.872) | <0.001 |
|  | PV_only | 0.873 (0.814-0.931) | 0.478 |
|  | simple_fusion | 0.865 (0.806-0.924) | 0.101 |
|  | mean_pool | 0.875 (0.815-0.935) | 0.429 |
|  | max_pool | 0.852 (0.789-0.914) | 0.043 |
| EVC2 | LFM-Seq | 0.795 (0.724–0.860) | — |
|  | AP_only | 0.781 (0.711-0.852) | 0.572 |
|  | PV_only | 0.782 (0.713-0.851) | 0.594 |
|  | simple_fusion | 0.797 (0.729-0.865) | 0.921 |
|  | mean_pool | 0.800 (0.731-0.869) | 0.782 |
|  | max_pool | 0.796 (0.726-0.866) | 0.967 |

Note. All ablations were performed with the LFM-Seq. The AP_only and PV_only use a single imaging phase, AP_only refers to arterial-phase input only and PV_only refers to portal venous-phase input only; simple fusion replaces cross-phase attention with direct concatenation; mean_pool and max_pool replace attention-based multiple instance learning (ABMIL) with mean or max pooling of patch features. AUCs were computed on the best checkpoint per task; 95% CIs and P values were obtained with the DeLong method, comparing each variant with the LFM-Seq model on the same cohort.

**Table S19. Incremental value of the LFM-Seq risk score over the clinicopathological model.**

| **Cohort** | **Time (months)** | **IDI (95% CI)** | **IDI *P* value** | **NRI (95% CI)** | **NRI *P* value** |
| --- | --- | --- | --- | --- | --- |
| EVC1 | 6 | 0.139  (0.059-0.232) | <0.001 | 0.357  (0.164-0.507) | <0.001 |
|  | 12 | 0.143  (0.065-0.227) | <0.001 | 0.365  (0.196-0.517) | <0.001 |
|  | 24 | 0.159  (0.081-0.240) | <0.001 | 0.464  (0.257-0.613) | 0.004 |
|  | 36 | 0.169  (0.086-0.262) | <0.001 | 0.602  (0.348-0.729) | <0.001 |
| EVC2 | 6 | 0.051  (0.006-0.120) | 0.024 | 0.265  (0.026-0.440) | 0.032 |
|  | 12 | 0.052  (0.007-0.117) | 0.032 | 0.269  (0.055-0.433) | 0.032 |
|  | 24 | 0.040  (0.002-0.115) | 0.040 | 0.220  (0.041-0.391) | 0.032 |
|  | 36 | 0.039  (0.000-0.120) | 0.052 | 0.213  (-0.001-0.415) | 0.052 |

Note. IDI = integrated discrimination improvement; NRI = net reclassification improvement (continuous). The reference model is the clinicopathological model; the fusion model adds the LFM-Seq risk score; CI = confidence interval.

**Table S20. Region-level overlap of high-contribution regions with the in-tumor and extended-tumor region for MVI assessment.**

| **Phase** | **Cohort** | **In-tumor overlap,**  **median (IQR)** | **Extended-tumor overlap, median (IQR)** |
| --- | --- | --- | --- |
| AP | IVC | 0.993 (0.662-1.000) | 1.000 (0.921-1.000) |
|  | EVC1 | 1.000 (0.753-1.000) | 1.000 (0.896-1.000) |
|  | EVC2 | 1.000 (0.738-1.000) | 1.000 (0.971-1.000) |
|  | All | 1.000 (0.741-1.000) | 1.000 (0.920-1.000) |
| PV | IVC | 0.675 (0.000-1.000) | 0.925 (0.000-1.000) |
|  | EVC1 | 0.241 (0.000-0.825) | 0.604 (0.000-0.960) |
|  | EVC2 | 0.362 (0.000-0.668) | 0.727 (0.000-0.950) |
|  | All | 0.362 (0.000-0.883) | 0.747 (0.000-0.979) |

Note. Values are reported as median (interquartile range [IQR]), where IQR represents the 25th–75th percentile range. The extended-tumor region was defined by expanding the tumor mask outward by 10 pixels.

**Table S21. Region-level overlap of high-contribution regions with the in-tumor and extended-tumor region for pathological grade assessment.**

| **Phase** | **Cohort** | **In-tumor overlap,**  **median (IQR)** | **Extended-tumor overlap, median (IQR)** |
| --- | --- | --- | --- |
| AP | IVC | 0.557 (0.254-0.882) | 0.760 (0.482-0.981) |
|  | EVC1 | 0.649 (0.350-0.820) | 0.841 (0.641-0.969) |
|  | EVC2 | 0.797 (0.509-0.939) | 0.941 (0.760-1.000) |
|  | All | 0.674 (0.309-0.917) | 0.886 (0.546-1.000) |
| PV | IVC | 0.526 (0.060-0.761) | 0.764 (0.121-0.965) |
|  | EVC1 | 0.654 (0.312-0.839) | 0.814 (0.582-0.996) |
|  | EVC2 | 0.787 (0.371-0.880) | 0.901 (0.609-1.000) |
|  | All | 0.618 (0.285-0.866) | 0.841 (0.516-0.992) |

Note. Values are reported as median (interquartile range [IQR]), where IQR represents the 25th–75th percentile range. The extended-tumor region was defined by expanding the tumor mask outward by 10 pixels.
